# A *Toxoplasma gondii* putative amino acid transporter localizes to the plant-like vacuolar compartment and controls parasite extracellular survival and stage differentiation

**DOI:** 10.1128/msphere.00597-23

**Published:** 2023-12-05

**Authors:** Federica Piro, Silvia Masci, Geetha Kannan, Riccardo Focaia, Tracey L. Schultz, Pariyamon Thaprawat, Vern B. Carruthers, Manlio Di Cristina

**Affiliations:** 1 Department of Chemistry, Biology and Biotechnology, University of Perugia, Perugia, Italy; 2 Department of Microbiology and Immunology, University of Michigan Medical School, Ann Arbor, Michigan, USA; University at Buffalo, Buffalo, New York, USA

**Keywords:** *Toxoplasma gondii*, bradyzoite differentiation, extracellular survival, amino acid transporters, arginine, SLC38A9, TORC complex

## Abstract

*Toxoplasma gondii* is a protozoan parasite that infects a broad spectrum of hosts and can colonize many organs and cell types. The ability to reside within a wide range of different niches requires substantial adaptability to diverse microenvironments. Very little is known about how this parasite senses various milieus and adapts its metabolism to survive, replicate during the acute stage, and then differentiate to the chronic stage. *T. gondii* possesses a lysosome-like organelle known as the plant-like vacuolar compartment (PLVAC), which serves various functions, including digestion, ion storage and homeostasis, endocytosis, and autophagy. Lysosomes are critical for maintaining cellular health and function by degrading waste materials and recycling components. To supply the cell with the essential building blocks and energy sources required for the maintenance of its functions and structures, the digested solutes generated within the lysosome are transported into the cytosol by proteins embedded in the lysosomal membrane. Currently, a limited number of PLVAC transporters have been characterized, with TgCRT being the sole potential transporter of amino acids and small peptides identified thus far. To bridge this knowledge gap, we used lysosomal amino acid transporters from other organisms as queries to search the *T. gondii* proteome. This led to the identification of four potential amino acid transporters, which we have designated as TgAAT1-4. Assessing their expression and sub-cellular localization, we found that one of them, TgAAT1, localized to the PLVAC and is necessary for normal parasite extracellular survival and bradyzoite differentiation. Moreover, we present preliminary data showing the possible involvement of TgAAT1 in the PLVAC transport of arginine.

**IMPORTANCE:**

*Toxoplasma gondii* is a highly successful parasite infecting a broad range of warm-blooded organisms, including about one-third of all humans. Although *Toxoplasma* infections rarely result in symptomatic disease in individuals with a healthy immune system, the incredibly high number of persons infected, along with the risk of severe infection in immunocompromised patients and the potential link of chronic infection to mental disorders, makes this infection a significant public health concern. As a result, there is a pressing need for new treatment approaches that are both effective and well tolerated. The limitations in understanding how *Toxoplasma gondii* manages its metabolism to adapt to changing environments and triggers its transformation into bradyzoites have hindered the discovery of vulnerabilities in its metabolic pathways or nutrient acquisition mechanisms to identify new therapeutic targets. In this work, we have shown that the lysosome-like organelle plant-like vacuolar compartment (PLVAC), acting through the putative arginine transporter TgAAT1, plays a pivotal role in regulating the parasite’s extracellular survival and differentiation into bradyzoites.

## INTRODUCTION

Among apicomplexans, *Toxoplasma gondii* is an exceptionally adaptable and successful parasite. Its ability to infect a wide variety of hosts, ranging from mammals to birds, is a testament to its versatility. Furthermore, this parasite has evolved strategies that allow it to colonize various organs and cell types within its many hosts, which is a key factor in its ability to persist and propagate. Thus, the adaptability of *T. gondii* is crucial for its survival in all these diverse microenvironments, and as an obligate intracellular parasite, it faces unique challenges for nutrient acquisition and regulation due to its dependency on host cell resources. Furthermore, *T. gondii* needs to adapt and temporarily survive in the extracellular milieu before invading a new host cell. How this parasite adapts its metabolism to respond to these drastic changes remains poorly understood. *T. gondii* possesses a lysosome-like organelle termed the plant-like vacuolar compartment (PLVAC) that plays multiple functions as a digestive organelle, being involved in ion storage and homeostasis, endocytosis, and autophagy (1). The PLVAC undergoes dynamic morphological changes throughout the parasite’s lytic cycle. In the extracellular and early invasion stages, the organelle presents as a singular, large vacuole. However, during intracellular replication, it undergoes fragmentation (2, 3). The PLVAC expresses various pumps and transporters facilitating the sequestration of potentially harmful ions such as zinc, calcium, and protons, among others. Additionally, it contains essential components like the vacuolar-H+-ATPase, the vacuolar-H+-pyrophosphatase (V-H+-PPase, TgVP1), and aquaporins. This organelle also appears to have homeostatic and calcium storage functions in the extracellular stage of the parasite. In fact, research on TgVP1 found that Δ*vp1* parasites are more sensitive to extracellular ionic concentrations and hyperosmotic or hypo-osmotic stress (2, 4). The hypothesis arising from these studies suggests that TgVP1 plays a key role in the survival of parasites under ionic stress during the short extracellular phase of the parasite and in preparing for the invasion of a new host cell. In another study, it was shown that the PLVAC zinc transporter TgZnT plays a crucial role in protecting extracellular tachyzoites from zinc toxicity (5). The role of the PLVAC in preserving parasites from toxicity of harmful metals is further supported by other recent research that identified a vacuolar iron transporter (VIT) that mediates iron detoxification in *Toxoplasma gondii* (6). Besides its homeostatic function, the PLVAC is a lysosome-like organelle that harbors acid hydrolases, including two canonical lysosomal cysteine proteases, cathepsin L and B (TgCPL and TgCPB) (7–9), and functions as a digestive organelle similar to mammalian lysosomes and plant lytic vacuoles (10). Moreover, Parussini et al., in their 2010 study (2), demonstrated the involvement of TgCPL in the proteolytic maturation process of pro-proteins targeted to micronemes.

As an intracellular parasite, *T. gondii* is heavily dependent on acquiring nutrients from the host to support its growth, both in the fast-replicating tachyzoite phase and in the slow-replicating bradyzoite phase. We have observed that *Toxoplasma* tachyzoites ingest host proteins and digest them within the PLVAC (11). When we interfered with this degradative process by disrupting TgCPL, it hinders tachyzoite replication and reduces the parasite’s virulence (11). Subsequent studies have made progress in understanding how host material is enclosed in vesicles (12), taken up by the parasite (13, 14), and transported to the PLVAC (15). Furthermore, the PLVAC plays a crucial role in recycling parasite organelles through autophagy during chronic infection (16, 17). Disrupting this recycling process, either by targeting TgCPL or by disrupting autophagy, results in a >100-fold decrease in brain cysts in chronically infected mice. Taken together, these studies show a multifaceted role of the PLVAC in various stages of the parasite’s lytic cycle, highlighting its essentiality for *Toxoplasma* infection. Despite the growing evidence that this organelle plays key roles in *Toxoplasma*, the content of the PLVAC is still poorly characterized. As a digestive organelle, the degradative material generated through PLVAC hydrolysis must be exported into the cytosol to facilitate its recycling for parasite metabolism. Only a limited number of PLVAC transporters have been characterized, with TgCRT being the sole potential transporter of amino acids and small peptides, based on its homology to PfCRT, identified thus far (18). Recent studies characterizing *T. gondii* Δ*crt* mutants have revealed a severely enlarged PLVAC (19, 20). This expansion of the organelle was particularly pronounced in bradyzoites and resulted in reduced parasite viability in the chronic form (19). Notably, deletion of TgCRT hindered the separation of the PLVAC from its precursor, the endosome-like compartment (ELC), during parasite replication in tachyzoites (21). These defects likely stem from the accumulation of solutes in the PLVAC due to the absence of TgCRT, leading to increased osmotic pressure and further enlargement of the organelle. Supporting this hypothesis was the observation that the PLVAC enlargement in Δ*crt* mutants was partially mitigated by inhibiting TgCPL, aligning with the suggested function of TgCRT as a transporter of proteolytic products. These studies underscore the pivotal role of TgCRT in regulating the size and integrity of the PLVAC by facilitating the movement of small molecules (osmolytes) from the organelle into the cytosol, thereby maintaining the PLVAC’s osmolarity.

The aim of the work presented herein was to identify additional PLVAC amino acid transporters and assess whether they play any role in parasite survival, growth, or differentiation. Here, we have identified bioinformatically four putative amino acid transporters that we named TgAAT1-4. One of them, TgAAT1, localizes to the PLVAC and is necessary for normal extracellular survival and stable inter-conversion between tachyzoites and bradyzoites in response to differentiation stimuli. Moreover, although supported by only preliminary data, TgAAT1 seems to be involved in arginine transport.

## RESULTS

### Identification of putative amino acid transporters in *T. gondii*

**Fig 1:**
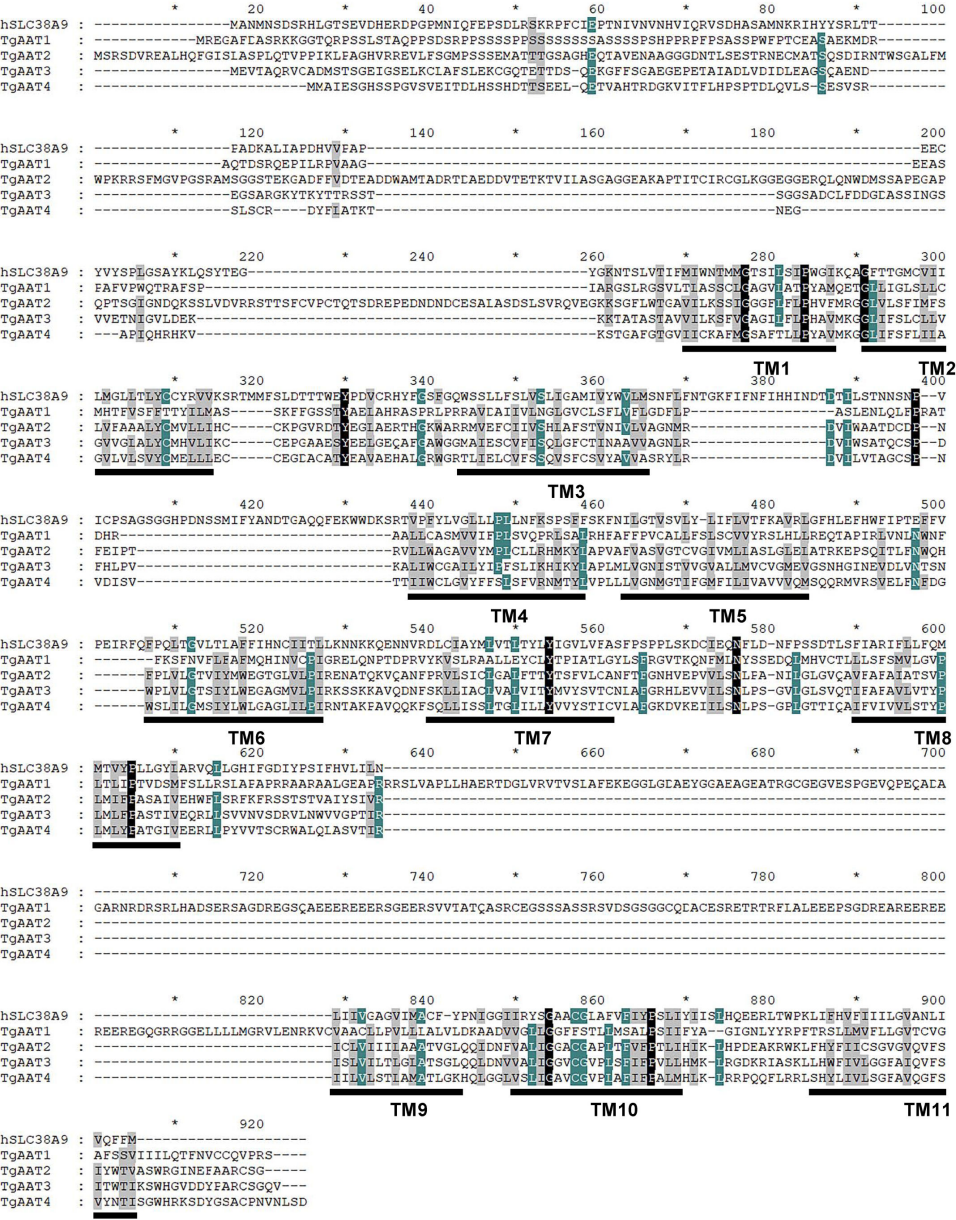
Multiple sequence alignment of SLC38A9 and putative amino acid transporters (TgAATs) using ClustalW software. Predicted transmembrane domains are underlined and numbered as TM1-11. Accession numbers of the proteins used in the alignment analysis are as follows: SLC38A9, *Homo sapiens*, and accession number NP_001336312.1. ToxoDB accession numbers for TgAATs are as follows: TgAAT1, TGME49_226060; TgAAT2, TGME49_227430; TgAAT3, TGME49_227570; and TgAAT4, TGME49_227580.

The *Toxoplasma* PLVAC serves numerous vital biological functions, one of which involves protein degradation and the subsequent release of the resulting digestion products, namely, amino acids and small peptides, into the cytosol. This process contributes to the pool of resources available for the various metabolic processes necessary for parasite survival. The efflux of amino acids from the PLVAC necessitates specialized transporters. Currently, TgCRT stands as the sole identified PLVAC transporter potentially involved in this process. This suggests the likelihood of the existence of other transporters that remain to be uncovered. To address this, we interrogated the *Toxoplasma* genome database ToxoDB (22) using known lysosomal or vacuolar amino acid transporters, such as human SLC38A9 or any yeast amino acid vacuolar transporters (Avt), as queries to identify putative *T. gondii* homologs. These searches always returned three hits that displayed modest similarity to the query but were annotated as amino acid transporters (Fig. S1). The three genes encoding these proteins (TGME49_227430, TGME49_227570, and TGME49_227580) are all located on chromosome X. TGME49_227570 and TGME49_227580 are adjacent to one another in a head-to-tail arrangement, whereas TGME49_227430 is ~30 kbp away and in the opposite orientation. The similarity and close linkage of these genes suggest that they arose by gene duplication from a common progenitor. A fourth hit displaying a very weak similarity with SLC38A9 and other yeast vacuolar proteins (Fig. S1), TGME49_226060, was also annotated as an amino acid transporter and is likewise located on chromosome X but ~2 million base pairs away from the other three putative genes. TGME49_226060 also displayed moderate similarity (19.0% identity; Fig. S1) to the *Plasmodium falciparum* amino acid transporter PfAAT1 but showed good superimposition of their Alphafold2-predicted three-dimensional (3D) structures (Fig. S1). Due to this homology, the protein encoded by TGME49_226060 was named TgAAT1, and TGME49_227430, TGME49_227570, and TGME49_227580 were named TgAAT2-4, respectively. Despite low amino acid conservation with SLC38A9, structural analysis of the TgAAT proteins using Alphafold2, TOPCONS, and I-TASSER software suggested the presence of 11 transmembrane domains that can be aligned by ClustalW with those of SLC38A9 (Fig. 1). The DALI server identified SLC38A9 as the best 3D superimposed structure for all the four TgAAT proteins, supporting the annotation of these proteins as amino acid transporters, which prompted us to characterize these integral membrane proteins further.

### TgAAT1 is a resident protein of the *T. gondii* PLVAC

**Fig 2:**
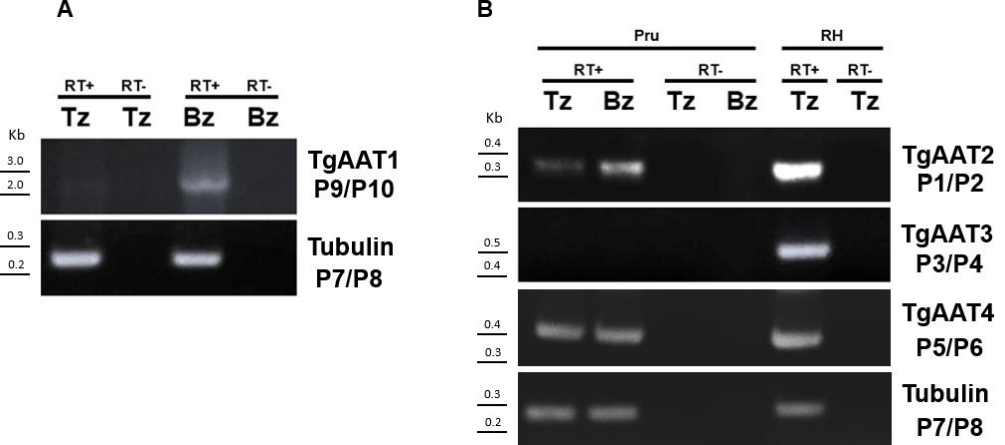
Semiquantitative RT-PCR analysis of TgAAT expression in either the tachyzoite or bradyzoite stages. (A and B) RNA isolated from *in vitro* tachyzoites (Tz) or bradyzoites (Bz) was reverse transcribed and used for PCR analysis using primers P9/P10 for TgAAT1 (panel A), P1/P2 for TgAAT2 (top panel B), P3/P4 for TgAAT3 (middle panel B), and P5/P6 for TgAAT4 (bottom panel B). Tubulin was amplified using primers P7/P8 (last of both panels A and B). Positive (“RT+”) and negative (“RT–”) signs indicate RNAs with or without reverse transcriptase, respectively. The representative agarose gel electrophoresis of RT-PCR products showed the expected bands of sizes: 100, 380, 400, 500, and 250 bp for TgAAT1, TgAAT2, TgAAT3, TgAAT4, and tubulin, respectively.

**Fig 3:**
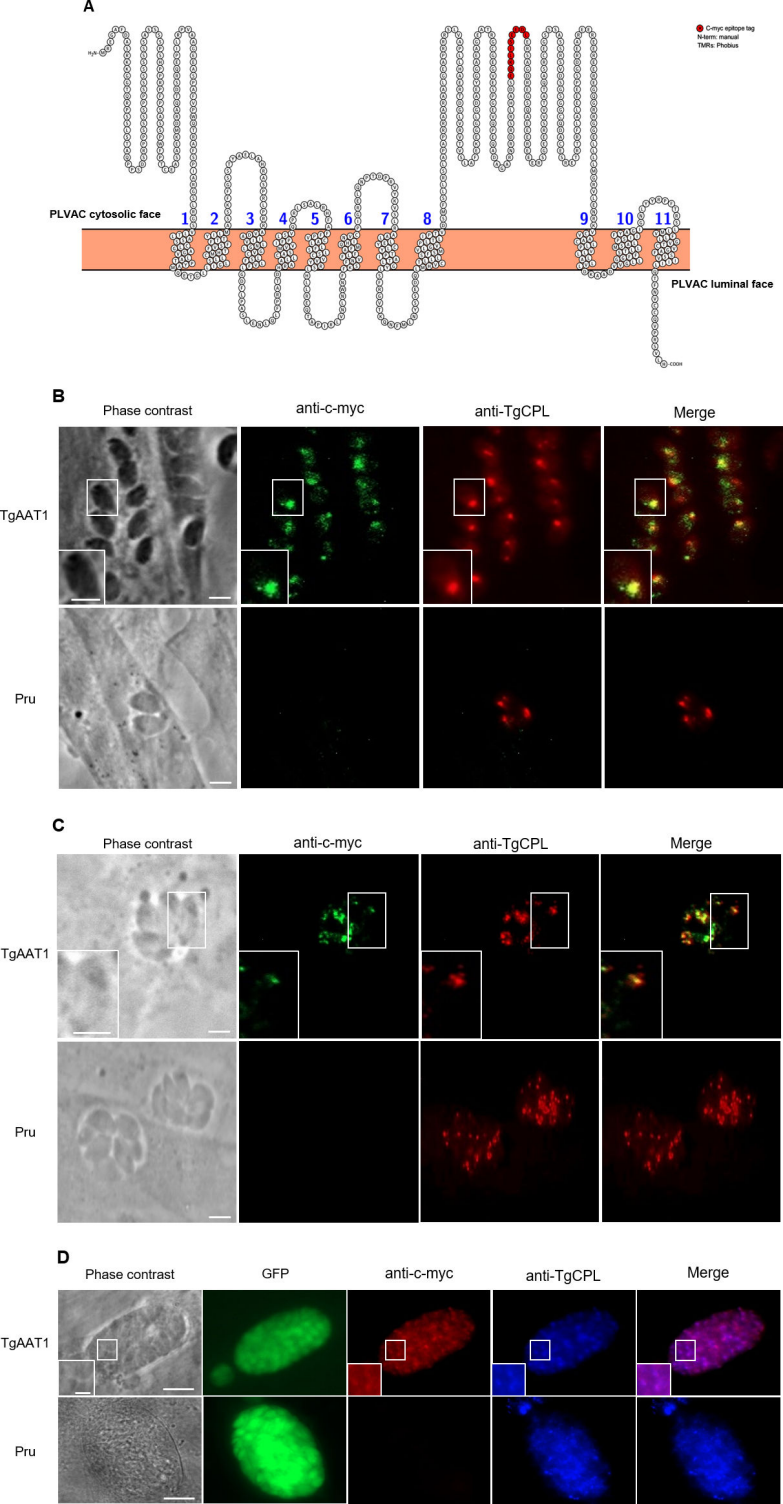
Analysis of TgAAT1 sub-cellular localization indicates that the protein is a resident on the PLVAC. (**A**) TgAAT1 was internally tagged with the c-myc epitope. Protter analysis (https://wlab.ethz.ch/protter/#) of the TgAAt1 amino acid sequence showing the integration of the c-myc tag in the loop between the predicted eighth and ninth transmembrane domains. (**B**) Intracellular TgAAT1 tachyzoites were stained with anti-c-myc (green) and anti-TgCPL (red) to mark the PLVAC (scale bar = 10 µm). TgAAT1 and TgCPL co-localization indicated that TgAAT1 is a resident on the PLVAC (inset, scale bar = 2 µm). (**C**) Intracellular replicating TgAAT1 tachyzoites were stained with anti-c-myc (green) and anti-TgCPL (red) to mark the PLVAC (scale bar 10µm). TgAAT1 and TgCPL co-localization indicated that TgAAT1 is resident on the PLVAC also during parasite replication (inset, scale bar 2µm). (D) TgAAT1 staining (red) of *in vitro* bradyzoites overlapped with that of TgCPL (blue), confirming the localization on the PLVAC (scale bar = 20 µm). Enhanced green fluorescent protein (EGFP) is expressed in bradyzoites of all strains derived from the parental PruΔ*ku80/Sluc*.

To determine the expression profile of the four TgAAT genes, we performed a series of semiquantitative RT-PCR experiments using RNA extracted from type II PruΔ*ku80/Sluc* strain (16) (hereafter called Pru) tachyzoites or *in vitro* differentiated bradyzoites. TgAAT2 and TgAAT4 were expressed at similar levels in both stages, while TgAAT1 showed low expression in tachyzoites and higher expression in bradyzoites (Fig. 2). TgAAT3 was not detected in Pru tachyzoites or bradyzoites but was expressed in type I RHΔ*ku80*Δ*hxprt* strain tachyzoites (Fig. 2B). Based on their expression in Pru, we endogenously tagged TgAAT1, TgAA2, and TgAAT4 with four copies of the c-myc epitope (4× c-myc) at their N- or C-terminal ends (Fig. S5). Disappointingly, none of the tagged strains were positive by immunofluorescence (IFA) or western blotting (WB) (data not shown) in both tachyzoites and bradyzoites. Failure to detect the four proteins could be explained by either low expression or cleavage of tags, particularly for the C-terminus with its predicted orientation toward the hydrolytic lumen of the PLVAC. To account for both potential explanations, we generated vectors to transiently overexpress each protein with a single N-terminal c-myc epitope. For TgAAT2 and TgAAT4, we again failed to detect expression by IFA or WB, possibly due to tight regulation of translation, poor protein stability, or loss of the c-myc epitope due to N-terminal processing. However, transient overexpression of TgAAT1 showed that it occupies the PLVAC based on co-localization with the PLVAC marker cathepsin protease L (TgCPL) (Fig. S6A and B). Furthermore, WB analysis of tachyzoites transiently transfected with c-myc TgAAT1 showed a band of the expected molecular weight of ⁓75 kDa (Fig. S6C). Since overexpression of TgAAT1 resulted in an appreciable signal by both IFA and WB, we generated a new strain in Pru where the *TgAAT1* promoter was replaced with that of *TgSAG1* together with adding a c-myc epitope at the N-terminus end of TgAAT1 (Fig. S6D through F). The higher expression that resulted from the stronger *TgSAG1* promoter confirmed the PLVAC localization of TgAAT1 by IFA (Fig. S6G). We also corroborated the PLVAC localization of TgAAT1 by endogenous insertion of a c-myc epitope (Fig. S7) into a large loop between the eighth and ninth transmembrane domains oriented toward the cytosolic face of the PLVAC (Fig. 3A through C). TgAAT1 staining in this latter strain was also detected in *in vitro* cysts after 1 week of differentiation induced by alkaline media (Fig. 3D). Collectively, these findings indicate that TgAAT1 localizes to the PLVAC, which prompted us to focus our investigation on it for the remainder of the study.

### TgAAT1 plays a key role in extracellular tachyzoite survival

**Fig 4:**
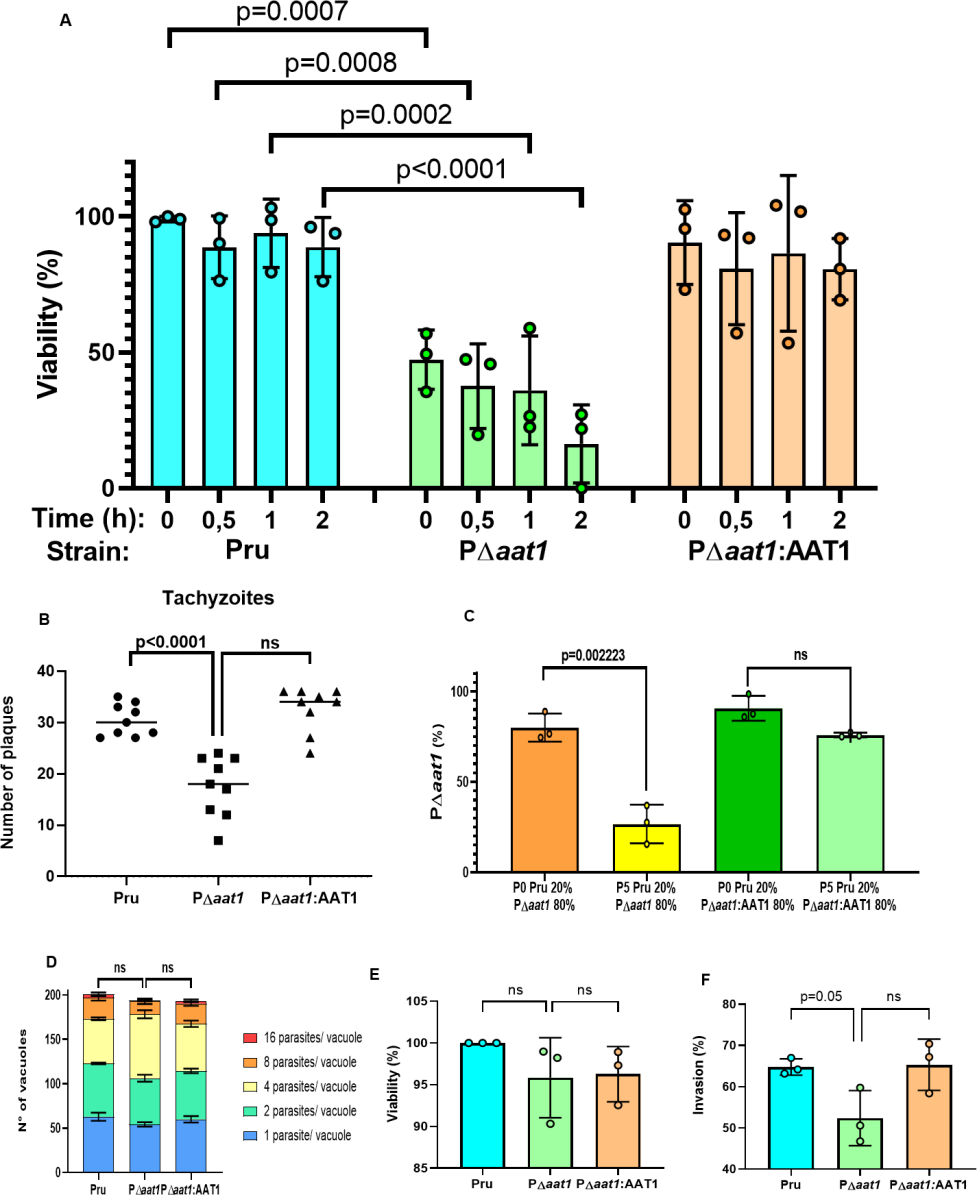
Phenotypic analysis of TgAAT1 tachyzoites. (**A**) TgAAT1 promotes the survival of tachyzoites upon egress from host cells. Tachyzoites of Pru, P∆*aat1*, and PΔ*aat1*:AAT1 were mechanically liberated from human foreskin fibroblast (HFF) cells, and 10^6^ parasites of each strain were incubated for 0, 0.5, 1, and 2 h in D10 media at 37°C and 5% CO_2_. After extracellular incubation, tachyzoite viability was assessed by propidium monoazide-quantitative polymerase chain reaction (PMA/qPCR) analysis. One-way analysis of variance (ANOVA) with Holm-Sidak’s multiple comparisons was used to compare the medians of data combined from three biological replicates. (**B**) Plaque assay of Pru, P∆*aat1*, and PΔ*aat1*:AAT1 tachyzoites showed that deletion of TgAAT1 resulted in a reduced number of plaques compared to both the parental and complement strains. Unpaired Student’s *t*-test was used to compare the medians of data combined from three biological replicates. (**C**) Competition assay to compare the fitness of the P∆*aat1* and complement tachyzoites with that of the Pru strain. HFF monolayers grown in T25 flasks were infected with 10^6^ tachyzoites of a mixture of either parental and PΔ*aat1* strains or parental and PΔ*aat1*:AAT1 strains at a ratio of 1:4. Parasites were grown for five passages and then harvested, purified, and used for qPCR analysis to calculate the ratio between the two mixed strains. The presence of the dihydrofolate reductase (DHFR) selection cassette in the P∆*aat1* and PΔ*aat1*:AAT1 strains but not in the parental strain was exploited to selectively amplify only the gDNA of these strains using primers binding to the tubulin promoter and DHFR cDNA of the selection cassette (see Table S1). Primers binding to the tubulin gene were used to normalize gDNA quantity since they amplified the gDNA of all three strains. The results showed the inversion of the 1:4 ratio in 4:1 after five passages of the only mixture parental/P∆*aat1*. The graph represents means ± SD from three independent experiments. Unpaired Student’s *t*-test was performed. (**D**) TgAAT1 ablation did not cause any replication defects. Parasites were cultured in monolayer HFF cells, and samples were collected at 24 h post-infection, fixed, stained with DAPI (4′,6-diamidino-2-phenylindole) and anti-TgSAG1, and quantified by fluorescence microscopy. At least 100 vacuoles were counted from six different fields of view. The percentages of different replication stages in the population for each strain were plotted. The results represent means ± SD from three independent experiments. Two-way ANOVA with Dunnett’s multiple comparisons was performed. p/v, parasites/vacuole. (**E**) PMA/qPCR viability assay of intracellular Pru, P∆*aat1*, and PΔ*aat1*:AAT1 tachyzoites. TgAAT1 ablation did not impact intracellular tachyzoite viability. The graph represents means ± SD from three independent experiments. One-way ANOVA with Holm-Sidak’s multiple comparisons was performed. (**F**) TgAAT1 ablation slightly impacts host cell invasion. Shown are the results of a red-green invasion assay of tachyzoites after 20 min of incubation with HFF cells. Parasites were stained as described in Materials and Methods. The graph represents means ± SD from three independent experiments. One-way ANOVA with Holm-Sidak’s multiple comparisons was performed.

To investigate the role of the TgAAT1 protein in the life cycle of *T. gondii*, we used CRISPR/Cas9 technology (23) to generate a TgAAT1 knockout (KO) strain, PΔ*aat1* (Fig. S1). A complement strain was also generated by re-expressing TgAAT1 as a cDNA driven by its own promoter and integrated into the tubulin locus (PΔ*aat1*:AAT1; Fig. S1). Characterization of these strains using classical assays such as plaque, invasion, replication, and competition assays allowed the identification of a strong defect in extracellular survival of PΔ*aat1*, which was rescued in the complement strain. Extracellular survival of the KO strain was impaired within 0.5 h of host cell extrusion, even if parasites were in rich media [Dulbecco’s modified Eagle’s medium (DMEM)], and was markedly reduced to 5%–30% after 2 h of extracellular incubation (Fig. 4A). The shorter tolerance to extracellular life of parasites lacking TgAAT1 may be responsible for the reduced number of plaques observed in the plaque assay (Fig. 4B). The parental strain is also outcompeting PΔ*aat1* in a co-infection assay (Fig. 4C). More specifically, co-infecting host cells with parental and PΔ*aat1* strains in a 1:4 ratio at passage 0 (P0) resulted in the inversion of this ratio to ~4:1 after P5, whereas no significant competition was seen between the parental and complement strains. Replication assays and intracellular tachyzoite viability showed no significant differences among the three strains (Fig. 4D and E), in line with similar plaque sizes (data not shown). Invasion assays revealed a modest phenotype, perhaps due to the reduced extracellular survival of PΔ*aat1* tachyzoites (Fig. 4F). Together, these findings indicate that TgAAT1 promotes extracellular survival of *T. gondii* tachyzoites.

### TgAAT1-deficient bradyzoites are less viable *in vitro* but show an increased cyst burden in chronically infected mice

**Fig 5:**
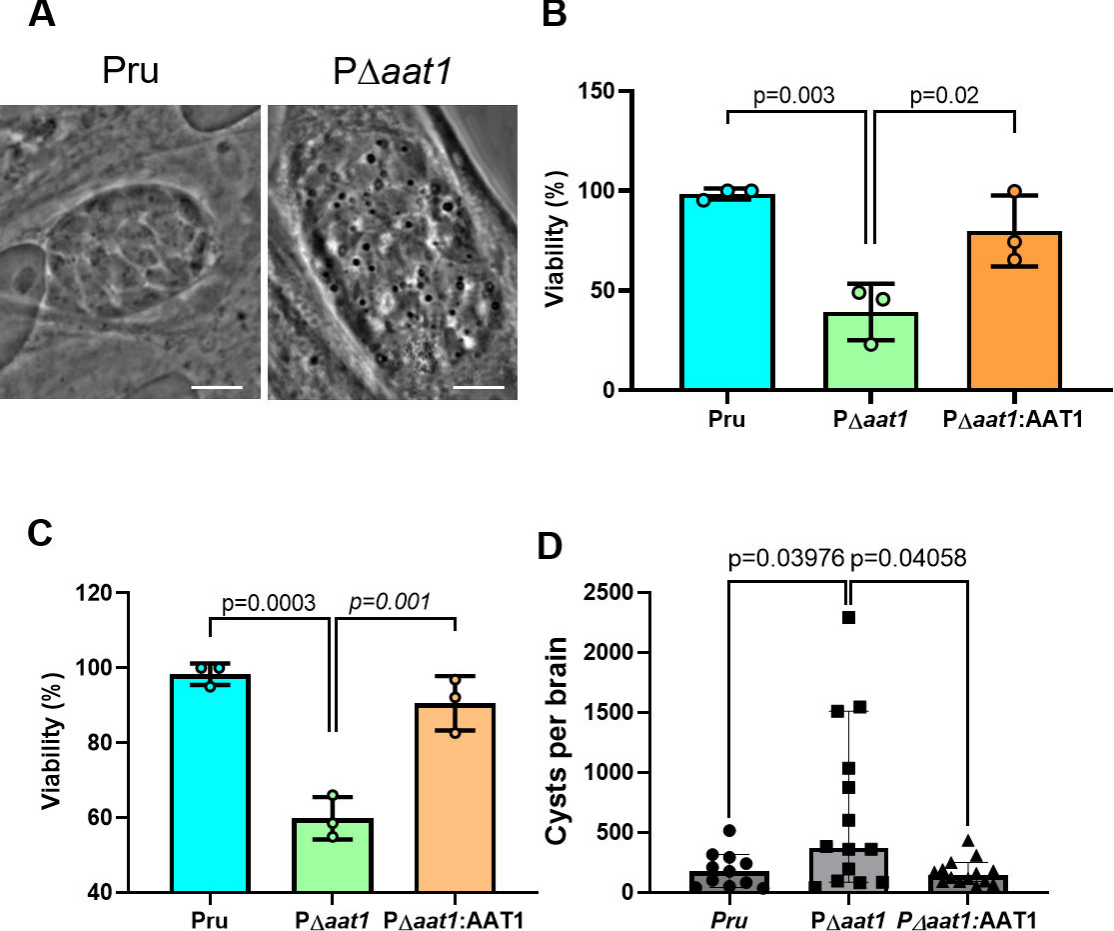
Deletion of the TgAAT1 gene led to significant changes in the formation, viability, and morphology of cysts containing bradyzoites. (**A**) Representative images of the parental or P∆*aat1 in vitro* cyst containing bradyzoites. Parasites were grown in alkaline media for 2 weeks, and then cysts were analyzed using optical microscopy under a ×100 objective. TgAAT1 ablation caused the alteration of morphology and the formation of translucent vacuoles and small dark dots in bradyzoites (scale bar = 10 µm). (**B and C**) TgAAT1-deficient bradyzoites displayed reduced viability. Pru, P∆*aat1*, and PΔ*aat1*:AAT1 tachyzoites were converted into bradyzoites by alkaline induction for either 1 week (**B**) or 2 weeks (**C**) and analyzed for viability using the PMA/qPCR assay. Error bars indicate the SD from three independent experiments. One-way ANOVA with Holm-Sidak’s multiple comparisons was performed. (**D**) Three groups composed of five CBA/J mice were infected with either the wild-type (parental), P∆*aat1*, or PΔ*aat1:*AAT1 strain. The experiment was repeated three times, resulting in a total of 15 mice for each group in the study. The graph depicts the number of cysts present in the brains of surviving mice from each group 5 weeks post-infection. The P∆*aat1* strain exhibited a higher number of cysts per brain compared to mice infected with both the wild-type and complement strains. This suggests that TgAAT1 ablation might have an impact on the parasite’s ability to form cysts within the host. The Mann-Whitney test was performed.

To determine whether ablation of TgAAT1 affected cyst formation, morphology, or viability, we analyzed the phenotype of PΔ*aat1* during the chronic stage of *T. gondii in vitro* and *in vivo*. Analysis of *in vitro* cysts showed that ablation of TgAAT1 caused altered morphology and formation of translucent vacuoles and small dark dots in bradyzoites (Fig. 5A). Although the nature of these alterations was not further investigated, PΔ*aat1* bradyzoites were ⁓40%–50% less viable than parental and complement strains after 1 or 2 weeks of *in vitro* conversion (Fig. 5B and C). Unexpectedly, CBA/J mice infected with 10^5^ PΔ*aat1* tachyzoites showed an ⁓10-fold increase in the brain cyst burden 5 weeks post-infection compared to those inoculated with the parental or complement strains (Fig. 5D). This *in vivo* analysis was repeated three times, and each experiment consisted of five mice per group infected with the same strain. Together, these findings suggest that although *in vitro* bradyzoites lacking TgAAT1 show altered morphology and viability, they conversely produced more brain cysts in infected mice.

### PΔ*aat1* showed unstable inter-conversion between tachyzoites and bradyzoites

**Fig 6:**
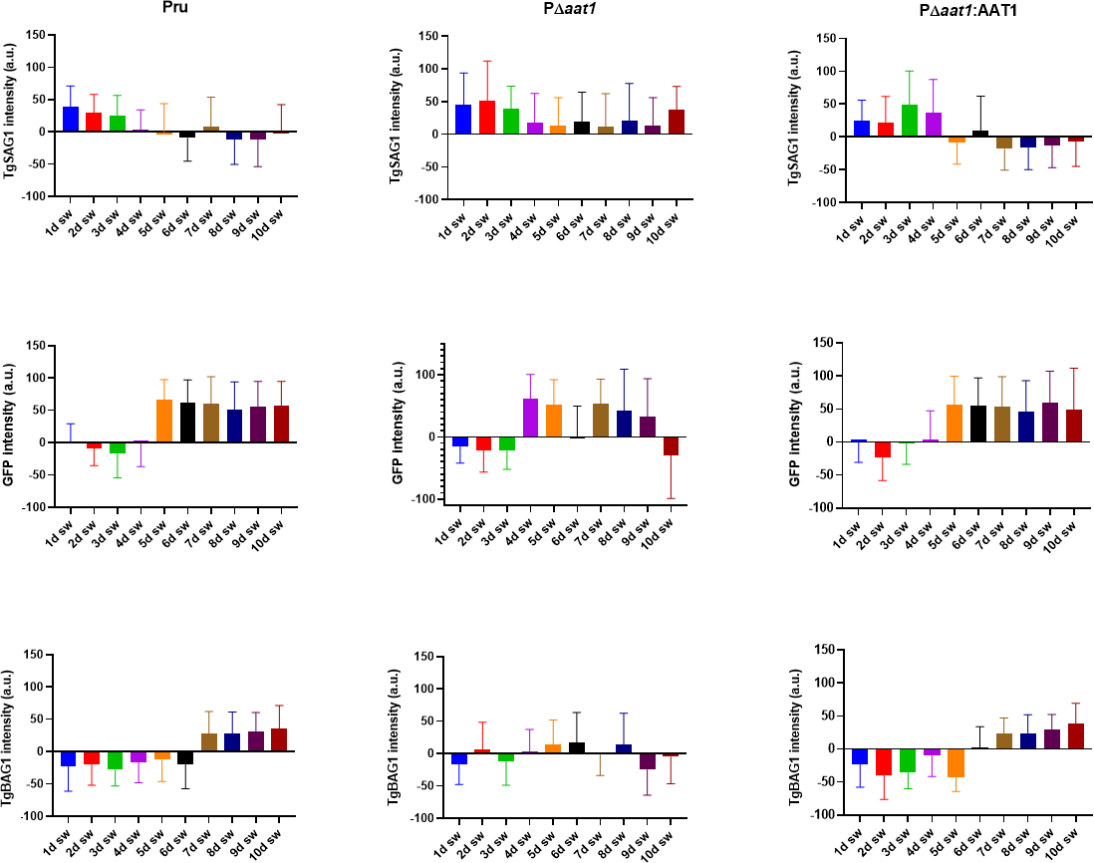
Ablation of the TgAAT1 gene leads to alterations in the tachyzoite-to-bradyzoite inter-conversion process. Bradyzoite conversion kinetics of Pru, P∆*aat1*, or PΔ *aat1:AAT1* strains were assessed in *in vitro* alkaline differentiation. Three groups of 20 HFF monolayers grown on coverslips were infected with 10^5^ tachyzoites of each strain. Tachyzoites were induced to convert into bradyzoites 24 h post-infection by replacing the D10 media with alkaline media. Two samples from each group were fixed every day from day 1 to day 10 post-conversion and stained with either rabbit anti-TgSAG1 or rabbit anti-TgBAG1 followed by anti-rabbit Alexa 594. The intensity of fluorescence for TgSAG1, TgBAG1, and EGFP was measured in 300 vacuoles from each slide by fluorescence microscopy. The results showed that deletion of the TgAAT1 gene caused changes in the behavior of the parasites, possibly affecting their ability to maintain the bradyzoite stage.

The brain cyst burden in infected mice is likely influenced by several factors, including the timing of tachyzoite-to-bradyzoite conversion and the strength of cues that the parasite uses for such conversion *in vivo*. The increased cyst burden observed in PΔ*aat1*-infected mice prompted us to assess the rate and efficiency of tachyzoite-to-bradyzoite inter-conversion. To this end, we measured the fluorescence intensity of parasite-containing vacuoles stained for either the tachyzoite marker TgSAG1 or the bradyzoite markers enhanced green fluorescent protein (EGFP) and TgBAG1 over the first 10 days of *in vitro* differentiation in alkaline medium. EGFP, which is driven by the LDH2 promoter, is an early bradyzoite marker, whereas TgBAG1 expression increases later during differentiation. Parental and complement strains showed the typical rate of conversion characterized by a gradual decrease in TgSAG1 during the first 4 days in alkaline medium, followed by the nearly complete loss of TgSAG1 and the appearance of EGFP and TgBAG1 from days 5 and 7 onward, respectively (left and right panels of Fig. 6). In contrast, the same analysis showed that ablation of TgAAT1 destabilized the inter-conversion between tachyzoites and bradyzoites (middle panels of Fig. 6). Notably, the tachyzoite marker TgSAG1 persisted at higher levels during the entire period of differentiation, including an apparent further increase on day 10 post-alkaline induction. Both the early and late bradyzoite markers, EGFP and TgBAG1, displayed an up and down expression with a drop in their signal intensity around days 9–10 post-induction, coinciding with the TgSAG1 peak. TgBAG1 in PΔ*aat1* parasites failed to reach the same intensity of signals observed in the parental and complement strains at any point during the 10 days in alkaline medium. Together, these findings suggest that PΔ*aat1* parasites show an unstable commitment to bradyzoite conversion under *in vitro* differentiation conditions.

### TgAAT1 function is linked to arginine

**Fig 7:**
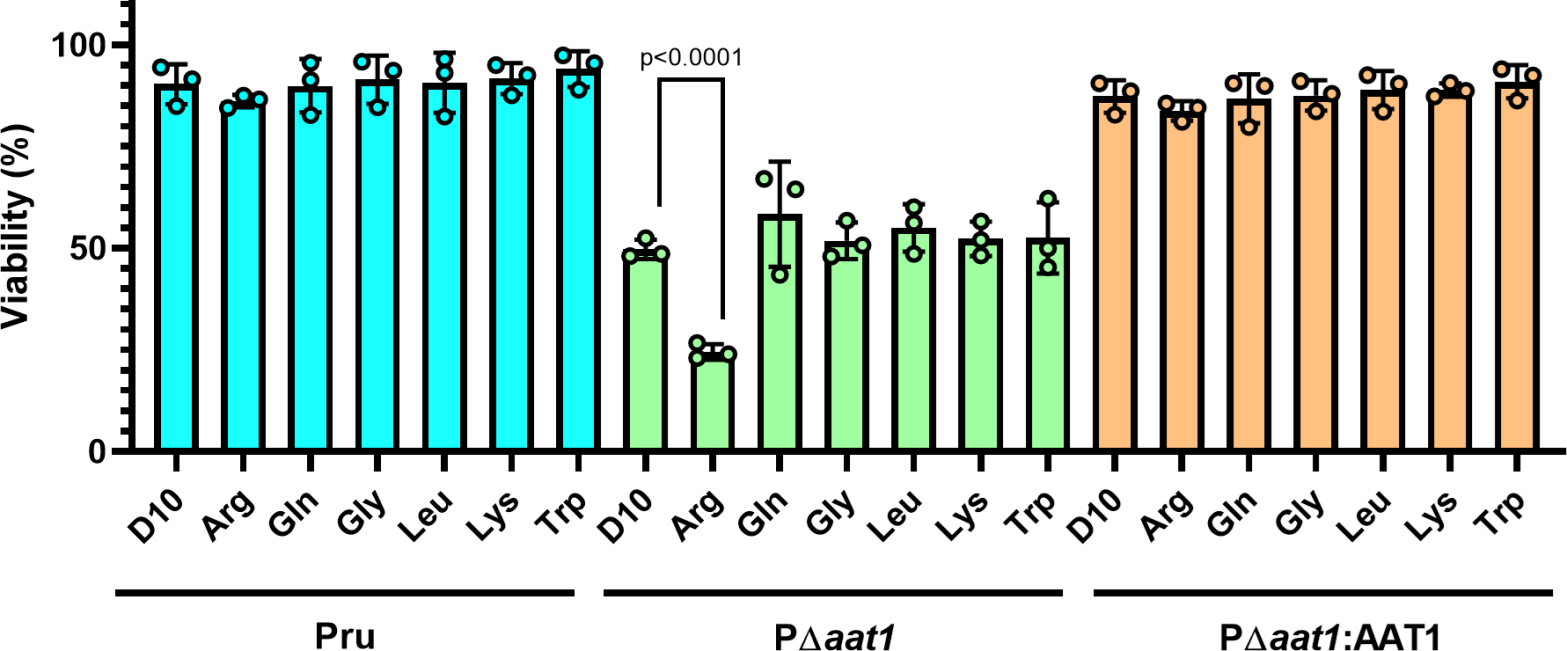
TgAAT1 showed increased mortality in the presence of esterified arginine. Extracellular Pru, P∆*aat1*, and PΔ*aat1*:AAT1 tachyzoites were incubated with normal RPMI media or RPMI media supplemented with one of the six different esterified amino acids for 1 h at 37°C and analyzed for viability using the PMA/qPCR method. Parasites were incubated with the following esterified amino acid concentrations: 25 mM Arg, 100 mM Gln, 5 mM Gly, 10 mM Leu, 10 mM Lys, and 10 mM Trp. Error bars indicate the SD from three independent experiments. One-way ANOVA with Dunnett’s multiple comparisons was performed.

**Fig 8:**
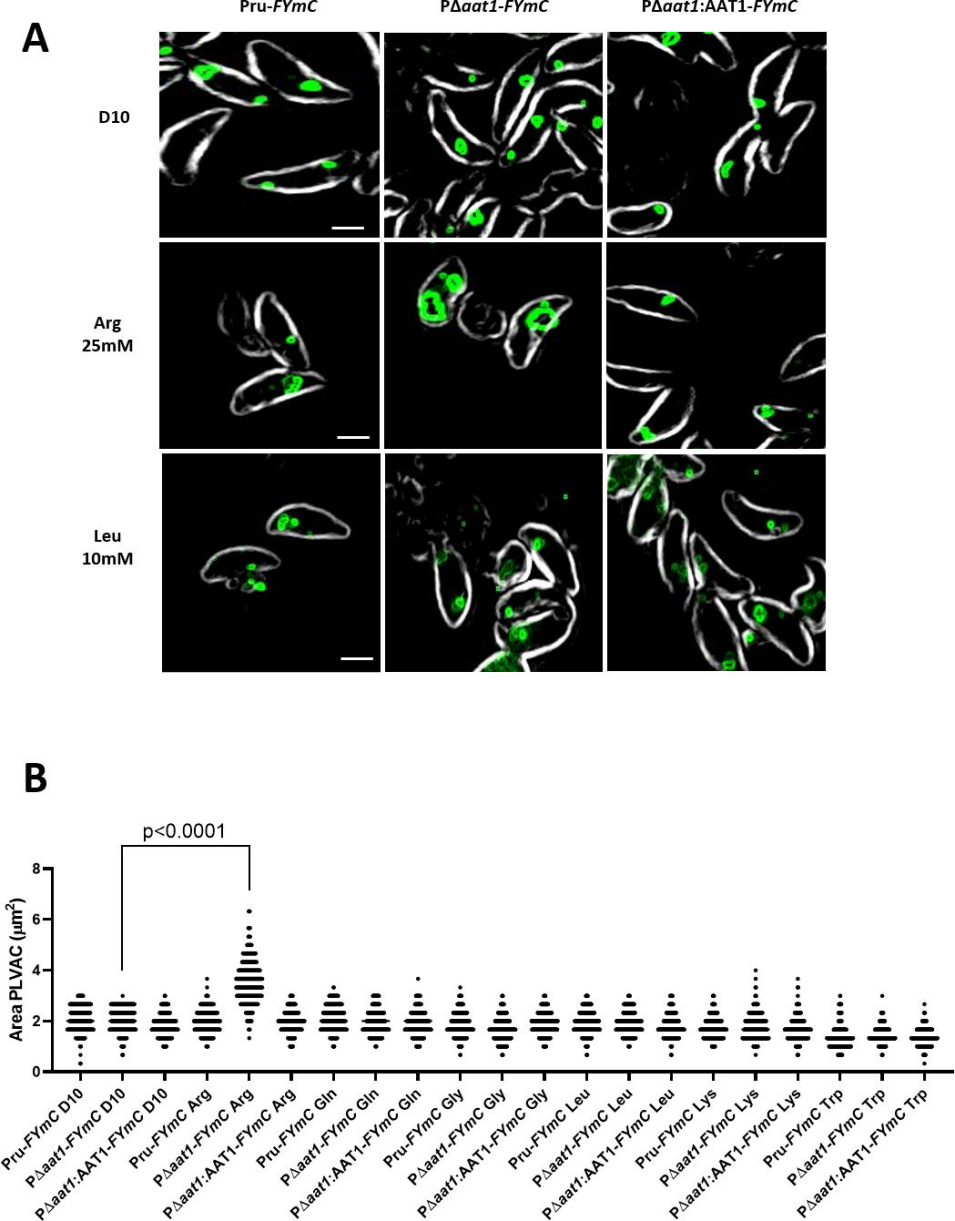
Arginine caused the enlargement of the PLVAC in parasites lacking the TgAAT1 gene. (**A**) Representative images of Pru*-FYmC*, P∆*aat1-FYmC*, and P∆*aat1:AAT1-FYmC* tachyzoites incubated with either D10, esterified arginine, or esterified leucine for 1 h and stained with anti-mCherry and anti-rabbit Alexa 594 (scale bar = 2 µm). (**B**) Area of the PLVAC of Pru*-FYmC*, P∆*aat1*-*FYmC*, and P∆*aat1:AAT1-FYmC* tachyzoites incubated with either D10 or one of the six esterified amino acids for 1 h. Parasites were incubated with the following esterified amino acid concentrations: 25 mM Arg, 100 mM Gln, 5 mM Gly, 10 mM Leu, 10 mM Lys, and 10 mM Trp. The area of 300 PLVACs was measured for each sample. One-way ANOVA with Holm-Sidak’s multiple comparisons was performed. Data are presented as means ± SD of three biological replicates, each with 300 PLVACs evaluated.

TgAAT1 is annotated as an amino acid transporter, and its 3D structure prediction and comparison with other proteins support this notion. Structural comparison of TgAAT1 using the DALI server identified the arginine transporter SLC38A9 as its best 3D superimposition hit. To investigate whether TgAAT1 was also capable of transporting arginine or other amino acids that are sensed to regulate cell proliferation, we used an esterified amino acid approach similar to that employed by Verdon et al. (24). Esterified amino acids are permeable to membranes and thus enter any sub-cellular compartment, including lysosomes. Lysosomal acid hydrolases remove the methyl-ester group, thereby causing the accumulation of the corresponding amino acid inside the lysosome, ultimately generating an osmotic effect (25). Cells can alleviate this stress by using an amino acid transporter(s) to efflux substrate amino acid(s) from the lysosome to the cytosol. Deleting a transporter can result in slower or absent efflux from the lysosome of the accumulated amino acid(s) that may become toxic for the cell that eventually dies. We selected six amino acids: arginine, glutamine, leucine, and lysine, because they are involved in TORC1 activation in other organisms (26, 27), and tryptophan and glycine as representative amino acids not involved in lysosomal nutrient sensing. Intracellular tachyzoites of the parental, TgAAT1 KO, and complement strains were mechanically extruded from human foreskin fibroblasts (HFFs), filter purified, and incubated for 1 h at 37°C in either normal RPMI media or RPMI media supplemented with one of the six selected esterified amino acids. The concentration of each esterified amino acid used in the assay was the highest tolerated, with no toxicity to the parental strain (Fig. S1). After incubation, the viability of parasites was measured by propidium monoazide-quantitative polymerase chain reaction (PMA/qPCR) assay adapted from other studies (28–37). As shown in Fig. 7, the P*Δaat1* strain exhibited approximately 50% viability across all samples due to the extracellular conditions except when incubated with esterified arginine, which caused an ⁓75% decrease in parasite viability. All esterified amino acids showed no significant effects in the parental or complement strains. This result suggested that the lack of TgAAT1 limited the efflux of arginine from the PLVAC, thereby reducing the capacity of KO parasites to rapidly compensate for the osmotic effect generated by overloading the organelle with this amino acid. To further test this possibility, we developed another assay also based on the esterified amino acid but with a different readout. Scerra et al. (38) showed that the reduced efflux of an amino acid from the lysosomes because of the lack of its transporter resulted in its accumulation within the organelle and consequent osmotic stress that caused the enlargement of the lysosomes. Thus, we applied a similar protocol to test whether any of the six selected amino acids caused the enlargement of the PLVAC of parasites lacking TgAAT1. To this end, we first endotagged the PLVAC protein TgFYVE (39) at its N-terminus with mCherry in the three strains (Fig. S1) and performed IFA using anti-mCherry antibodies to stain the PLVAC membrane where this protein localized (Fig. 8A). Exploiting this staining, we measured the area of 900 PLVACs of parental, PΔ*aat1*, and complement tachyzoites. This analysis showed that arginine was again the only amino acid of the six selected amino acids that caused a significant enlargement of the PLVAC in PΔ*aat1* compared to the other two strains (Fig. 8B). Collectively, these assays provide indirect evidence that TgAAT1 is necessary for the normal efflux of arginine from the PLVAC.

## DISCUSSION

While many aspects of *T. gondii* biology are now well characterized, there remains a gap in our understanding of the molecular mechanisms that govern its adaptation to varying environments, especially in terms of nutrient availability and how this impacts its survival, growth, and differentiation between different life stages. The PLVAC has been demonstrated to participate in various survival pathways, including metal detoxification (3, 5, 6), cell homeostasis (40), and autophagy (8, 16, 17). Of these, autophagy plays a crucial role in facilitating extracellular survival (41, 42) and cyst persistence (16, 17). Autophagy is a process in which cytoplasmic contents, including organelles, are encapsulated by membranes and subsequently degraded within lysosomes (43). Deletion or inhibition of TgCPL in bradyzoites compromised the PLVAC’s ability to digest proteins within the organelle, resulting in the accumulation of undigested autophagic material within the PLVAC and contributing to parasite death (16). While the exact reason why TgCPL-depleted bradyzoites perish remains unknown, these findings underscore the significance of the PLVAC as a central hub for the degradation of autophagic vesicles, possibly for organelle renewal or to compensate for a limited nutrient supply due to the physical barrier of the cyst wall restricting resource acquisition from the host. The efflux of digested material from the inside of the lysosomes toward the cytosol requires specific transporters. Currently, aside from TgCRT, whose transport specificity has not been extensively characterized but by similarity to PfCRT might transport small peptides, no specific PLVAC amino acid transporters have been identified. We then performed searches in ToxoDB using human lysosomal or yeast amino acid vacuolar transporters (avt) to identify additional putative PLVAC amino acid transporters. The results from these analyses consistently identified the same four proteins encoded by TGME49_226060 (TgAAT1), TGME49_227430 (TgAAT2), TGME49_227570 (TgAAT3), and TGME49_227580 (TgAAT4), although amino acid identity was modest and restricted to relatively small regions of these proteins. More convincing, structural analysis of all four proteins using the DALI server identified SLC38A9 as the best 3D structural homolog. However, despite N- and C-terminal tagging, we never detected signals for TgAAT2, TgAAT3, or TgAAT4 by IFA or WB, not even when overexpressed in transient transfection experiments. In contrast, TgAAT1 was clearly expressed in parasites transiently transfected with a vector carrying the coding cDNA under the control of the tubulin promoter and fused at the 5′-end with the c-myc sequence. TgAAT1 localized to the PLVAC, as its staining in IFA overlapped with that of the organelle marker TgCPL. However, endogenous tagging of the TgAAT1 N-terminus was not detectable unless the promoter was swapped with that of TgSAG1 to increase expression. Although IFA detection of endogenous internally tagged TgAAT1 was weak, it was sufficient to confirm the PLVAC localization in non-overexpressing parasites. Homology searches using TgAAT1 as the query identified orthologs in several apicomplexans, including *Plasmodium falciparum*, which expresses PfAAT1 (44). Recent work on PfAAT1 has revealed that it localizes to the digestive vacuole (DV) and that mutations of critical residues conferred parasite resistance to chloroquine, similar to PfCRT (45, 46). Despite the important role played by PfAAT1 in drug resistance, its transporter specificity has not been addressed so far.

Our work on TgAAT1 has allowed the identification of its role in promoting extracellular survival of *T. gondii* tachyzoites. Deletion of TgAAT1 markedly reduced tachyzoite tolerance to extracellular life, with decreased viability seen within 0.5 h of mechanical cellular extrusion. This phenotype may explain results obtained from the competition assay where parental parasites outgrew PΔ*aat1*, not the complement strain, when mixed and allowed to replicate in the same host cell monolayers. Whether TgAAT1 supports extracellular survival directly, e.g., by providing amino acids from PLVAC proteolysis, or indirectly, e.g., by working as a sensor that activates a survival pathway, remains to be determined and deserves further investigation. In contrast, the viability of intracellular tachyzoites was not affected by the lack of TgAAT1. Interestingly, *in vitro* PΔ*aat1* bradyzoites were ⁓50% less viable than parental and complement strains. Moreover, *in vitro* differentiated PΔ*aat1* bradyzoites showed an altered morphology and the presence of translucent vacuoles and small dark dots within their cytoplasm. Such morphologic changes are reminiscent of those seen in bradyzoites lacking TgCPL. Similarly, TgCPL proteolytic activity is not critical for tachyzoites but is more important for bradyzoite viability (16). However, the cyst burden of mice infected with PΔ*aat1* was 10-fold higher than that of parental and complement strains. Although this finding was in apparent contradiction with the reduced bradyzoite viability of PruΔ*aat1*, analyses of the rate and efficiency of tachyzoite-to-bradyzoite inter-conversion over 10 days indicated that the lack of PΔ*aat1* caused unstable differentiation, as we observed many bradyzoites converting back to tachyzoites. This stage instability may be responsible for the higher brain cyst burden observed in mice infected by PΔ*aat1*. Hypothetically, incomplete bradyzoite differentiation, with some of them converting back into tachyzoites, might be responsible for new waves of tachyzoite expansion in the brain before forming new cysts. Also, *T. gondii* might receive stronger cues to differentiate *in vivo*, thus potentially accounting for the eventual formation of more cysts in infected mice.

Furthermore, consistent with the putative role of amino acid transporters, we have applied an indirect but previously validated approach based on the use of esterified amino acid precursors (24, 38) to assess whether TgAAT1 can transfer any of the amino acids that are sensed by the nutrient-sensing machinery in other eukaryotes. We focused this analysis on the six amino acids, arginine, glutamine, leucine, and lysine, which are TORC1 activators in other organisms, along with tryptophan and glycine as representative amino acids not involved in the amino acid sensing pathway. Notably, arginine was the only amino acid that caused parasite death and PLVAC swelling.

Taken together, these data suggest that TgAAT1 plays a key role in extracellular parasite survival and differentiation since its ablation results in weaker extracellular tolerance and instability in maintaining the correct parasite form. In one scenario, TgAAT1 might provide amino acids to temporarily sustain *T. gondii* metabolism when parasites lack access to host-derived nutrients. Alternatively, TgAAT1 may work as a sensor that regulates parasite metabolism to respond to environmental changes. Although we have no direct evidence that TgAAT1 is a nutrient sensor, that TgAAT1 is necessary for normal and stable differentiation between tachyzoites and bradyzoites may be indicative of this function. Moreover, TgAAT1 may play a role in the efflux of arginine across the PLVAC membrane. This amino acid is recognized as a pivotal factor in regulating *T. gondii* differentiation, as evidenced by studies where the growth of tachyzoites in arginine-deficient media induced bradyzoite conversion (47).

In conclusion, our work represents a step toward identifying a new component of the PLVAC and underscores once more the central role played by the PLVAC in various aspects of parasite biology.

## MATERIALS AND METHODS

### Host cell and parasite cultures

Human foreskin fibroblasts were grown and maintained in Dulbecco’s modified Eagle’s medium containing 10% fetal bovine serum (Thermo Fisher) and 50 µg/mL penicillin-streptomycin (media called D10). All *T. gondii* strains were propagated *in vitro* by serial passage on HFF monolayers (48). *In vitro* tachyzoite-to-bradyzoite conversion was induced by exposing parasite cultures to pH 8.2 (49, 50).

### Generation of transgenic *T. gondii* strains

Pru*∆ku80Luc* (16) (herein termed Pru) was used as the parental strain to generate all transgenic lines used in this study. The parental strain expresses enhanced green fluorescent protein when converted into bradyzoites due to integration in its genome of an EGFP cassette expressing the fluorescent marker under the control of the LDH2 bradyzoite stage-specific promoter, as previously described (51). The TgAAT1 knockout and all tagging strains were generated using CRISPR/Cas9 technology as described in Rivera-Cuevas et al. (12). Guide RNAs (gRNAs) used for genome manipulations are listed in Table S1. Schematic representations of the genomic manipulation strategy and PCR validation of all strains generated are shown in the supplemental figures. Plasmids used to overexpress TgAAT proteins via transient transfection of Pru were generated by cloning *TgAAT* cDNAs in the pTub/CAT vector (52) by replacing the CAT gene using NEBuilder HiFi DNA Assembly technology (NEB E5520S). TgAAT cDNAs were amplified using forward primers containing the coding sequence for the c-myc tag to fuse the c-myc epitope at the N-terminus of the TgAAT proteins. Complementation of TgAAT1 (PΔ*aat1*:AAT1) was accomplished by integrating a plasmid carrying the TgAAT1 cDNA cloned downstream of 1,581 bp of the TgAAT1 sequence upstream of the initiation codon to drive transcription of these sequences, followed by 312 bp of the 3′UTR of the TgSAG1 gene and the BLE selection cassette. The plasmid was integrated into the *T. gondii* genome upstream of the tubulin gene by introducing into the complement plasmid a 1,300-bp fragment derived from the tubulin locus and linearization using *Pme*I to induce single crossover, as described in Kannan et al. (19). *T. gondii* transfections were performed as described previously (53). Correct integrations were confirmed by PCR analysis of single clones using a Phire Tissue Direct PCR Master kit (Thermo) (54).

### Immunoblotting and immunofluorescence

Immunoblotting and immunofluorescence were performed as described (12). Mouse anti-c-myc was diluted 1:1,000 and 1:100 in WB and IFA, respectively. Anti-TgCPL was used at 1:200 in IFA (16).

### Tachyzoite plaque assay

Intracellular tachyzoites were mechanically liberated and purified following standard procedures. Three hundred tachyzoites were added to HFF monolayers grown in six-well plates in triplicate or quadruplicate wells. Parasites were left undisturbed for 12 days at 37°C and 5% CO_2_. Plates were then stained with crystal violet, and plaques were counted.

### Competition assay

HFF monolayers were infected with 10^6^ parasites composed of a mixture of either two strains, Pru and P∆*aat1* or Pru and P∆*aat1:AAT1*, in a 1:4 ratio. Parasites were collected after five passages of monolayer lysis, and their genomic DNAs were analyzed by qPCR using the primers Tub-F1 and DHFR-R1 (listed in Table S1) designed to amplify 119 bp of the DHFR selection cassette (absent in Pru but present in P∆*aat1* and P∆*aat1:AAT1*). Amplification of the tubulin gene (present in all parasites) was used to normalize the gDNA quantity.

### PMA/qPCR assay

This new protocol was developed by modifying the PMA viability assay applied to bacteria and other eukaryotes (28–37). Exploiting the propidium monoazide (PMA) molecule, it is possible to discriminate dead from live cells. PMA is not permeable to membranes and thus enters only dead cells because membranes are damaged. Inside dead cells, PMA binds genomic DNA (gDNA). Exposure of these samples to 470-nm blue light covalently cross-links PMA to gDNA. This irreversible binding renders the gDNA not amplifiable by PCR. Therefore, only the gDNA of live parasites, not accessible to PMA binding, can be amplified from samples incubated with PMA and exposed to blue light. Variability due to loss of material during centrifugation steps was minimized by directly performing qPCR on aliquots of PMA-treated and non-treated parasites after adding the DNARelease Additive from the Phire Tissue Direct PCR Master kit (Thermo Fisher Scientific) without any gDNA purification.

### Intracellular tachyzoite viability assay

Intracellular tachyzoite viability assay was performed by mechanically liberating parasites from infected monolayers and purifying tachyzoites from cell debris by filtration through 3-µm filters. Thirty-nine microliters was immediately transferred to new tubes and incubated with or without 30 µM PMAxx (Biotium) for 15 min with shaking and in darkness. Incubation with PMAxx was followed by blue light exposure for 15 min using a Blue LED Device (PMA-Lite LED Photolysis Device Biotium #E90002). After blue light exposure, 1 µL of DNARelease Additive was added to each sample followed by incubation at 22°C for 4 min and at 98°C for 2 min in a PCR machine. One microliter of DNA from each sample was used to perform qPCR in 10 µL of total mix from POWERTRACK SYBR MM (Thermo) using 0.3 µM multilocus primers Tx9 and Tx11 (Table S1). PCR running parameters were set as follows: 95°C for 15 s and 60°C for 30 s for 40 cycles. Viability was calculated as follows:

- dCt_sample_ = Ct_(PMAxx-treated sample)_ − Ct_(untreated sample)_
- Fold change = 2^dCt^_sample_
- % viability = 100/fold change

### Extracellular tachyzoite viability assay

Extracellular tachyzoite viability assay was performed by mechanically liberating parasites from infected monolayers and purifying tachyzoites from cell debris by filtration through 3-µm filters and three washes with D0 (DMEM without FBS) to remove the D10 media. Extracellular parasites were resuspended in D0 at a concentration of 5 × 10^6^/mL, and 39 µL was analyzed using the same protocol described above and applied to intracellular tachyzoites.

### Bradyzoite viability assay

Bradyzoite viability assay was performed as follows: 1 × 10^2^ tachyzoites per well were used to infect HFF monolayers grown in 96-well plates. Twenty-four hours post-infection, tachyzoites were induced to convert into bradyzoites by replacing D10 media with alkaline media for 1 week or 2 weeks, refreshing the media daily. After conversion, infected monolayers were washed three times with HBSS, and bradyzoites were released from *in vitro* cysts by adding 33.2 µL of pre-warmed pepsin solution (0.026% pepsin in 170 mM NaCl and 60 mM HCl, final concentration) and through incubation at 37°C for 1 h (the HFF monolayer is degraded by this treatment). Reactions were stopped by adding 1 vol (33.2 µL) of 188 mM Na_2_CO_3_. Bradyzoite viability was performed by transferring 33.2 µL from each sample into two new 96-well PCR plates for PMA-treated and untreated samples and placing the 96-well plates on ice. PMA treatment was performed by adding 5.8 µL of 200 µM PMAxx to 33.2 µL of the PMA-treated samples, while the same volume of ddH_2_O was added to the untreated tubes. Samples were incubated for 15 min with shaking and in darkness and then exposed to blue light for 15 min using the PMA-Blue LED Device. One microliter of DNARelease Additive was added to each sample, followed by incubation at 22°C for 4 and 2 min at 98°C in a PCR machine. One microliter of each sample was used in qPCR to assess viability as described above for tachyzoites.

### Invasion assay

Invasion assay was performed as already described in Possenti et al. (55) by differential IFA using a red-green assay (56) by labeling exclusively attached (extracellular) tachyzoites in non-permeabilized samples with anti-TgSAG1, and after permeabilization with 0.2% Triton X-100, all parasites were stained with a rabbit anti-GAP45 antibody. Three hundred microscopic fields/well at ×100 magnification were examined. The data shown are representative of experiments performed in triplicate.

### Replication assay

Freshly egressed parasites were used to infect HFF monolayers grown on coverslips in six-well plates at a density of 10^6^ tachyzoites/well for 20 min. After four washes to remove uninvaded tachyzoites, the cultures were incubated for 24 h at 37°C and 5% CO_2_ prior to fixation with 4% PFA. Infected monolayers were permeabilized with 0.2% Triton X-100 and processed for IFA using rabbit anti-GAP45 and Alexa 594-conjugated anti-rabbit IgG antibodies. The number of parasites per vacuole was enumerated by examining 300 microscopic fields/well at ×100 magnification. The data shown are representative of experiments performed in triplicate.

### *In vivo* cyst viability

Mouse experiments were performed according to guidelines from the United States Public Health Service Policy on Humane Care and Use of Laboratory Animals. Animals were maintained in an AAALAC-approved facility, and all protocols were approved by the Institutional Animal Care and Use Committee of the University of Michigan School of Medicine, Ann Arbor, MI, USA (Animal Protocol PRO00010428; Animal Welfare Assurance no. A3114-01). CBA/J female mice (7 to 8 weeks old; Jackson Laboratories, Bar Harbor, ME, USA) were used in this study. Mice were injected intraperitoneally (i.p.) with 10^5^ purified tachyzoites in 200 µL of 1 × phosphate-buffered saline (PBS). Five weeks post-infection, mice were sacrificed according to university-approved protocols. Brains were harvested and homogenized in 1 mL of ice-cold PBS by syringing through a 20-gauge needle. Cysts were enumerated in 2 × 100 µL of the brain homogenate by fluorescence microscopy, and the total brain cyst numbers were calculated. Cyst burden data were pooled from three independent experiments.

### Bradyzoite time course

Freshly egressed tachyzoites were used to infect 20 HFF monolayers grown on coverslips for each Pru, P∆*aat1*, and P∆*aat1:AAT1* strain and converted into bradyzoites by alkaline induction 24 h post-infection. Two slides per strain were fixed every day from day 1 to day 10 post-alkaline induction to generate two series of time course conversion. One series for each strain was stained with a mouse anti-TgSAG1 antibody (1:100) followed by an Alexa 594-conjugated anti-mouse IgG antibody (1:1,000), and the second series was stained with a rabbit anti-TgBAG1 antibody (1:400) followed by an Alexa 594-conjugated anti-rabbit IgG antibody (1:1,000). Five hundred cyst images were collected to measure fluorescent intensity arbitrary units (a.u.) of TgSAG1, TgBAG1, and GFP staining. Because parasite vacuole/cyst images even when negative for a specific marker have a background that slightly changes from image to image, we have subtracted from the signal value of each marker the value obtained as the average of the signal intensity collected from 500 Pru-negative vacuoles on day 1 for TgBAG1 and EGFP signals, when these markers are not expressed, and on day 10 for TgSAG1, selecting negative cysts (some TgSAG1-positive tachyzoite vacuoles were still present even after 7–10 days of alkaline induction).

### Esterified amino acid viability assay

Intracellular parasites of the Pru, P∆*aat1*, and P∆*aat1:AAT1* strains were mechanically liberated from infected HFF monolayers by syringing through 26G needles, centrifuged at 800 × *g* for 10 min at 4°C, and then resuspended in 1.5 mL of D10. A total of 10^6^ tachyzoites of each sample were transferred to each well of a 96-well plate with a V bottom in a volume of 200 µL for each esterified amino acid or control analysis. After centrifuging the 96-well plate, parasites were resuspended in RPMI media where the amino acid to be tested was replaced with the esterified amino acid and then incubated at 37°C with 5% CO_2_ for 1 h. The quantity of each esterified amino acid supplemented to the RPMI media was selected as the highest that was not toxic to the parental strain. The viability of all samples was analyzed using the PMA/qPCR protocol described above.

### PLVAC size assay

Pru-*FYVE-mCherry* (Pru*-FYmC*), P∆*aat1-FYVE-mCherry* (P∆*aat1-FYmC*), and P∆*aat1:AAT1-FYVE-mCherry* (P∆*aat1:AAT1-FYmC*) parasites were collected using the same protocol for esterified amino acid viability assay. After incubation, extracellular parasites were settled on Cell-Tak (Fisher Scientific)-coated slides for 30 min at 4°C, fixed in 4% formaldehyde, and stained with a rabbit anti-mCherry antibody (1:500, Merck) followed by an Alexa 594-conjugated anti-rabbit IgG antibody (1:1,000). Three hundred images for each sample were acquired by focusing on the PLVAC signal on a Nikon TE2000-S Inverted Fluorescence Microscope equipped with a ×100 oil objective and processed using ImageJ software. PLVAC size was measured by defining the area of anti-mCherry immunofluorescence.

### Statistics

Data were analyzed using GraphPad Prism. For each data set, outliers were identified and removed using ROUT with a *Q* value of 0.1%. Data were then tested for normality and equal variance. If the data passed, one-way or two-way analysis of variance (ANOVA) was performed. If the data failed, a Mann-Whitney *U* test was performed.

## SUPPLEMENTAL MATERIAL

Supplemental Figures msphere.00597-23-s0001.pdf Fig. S1 to S10.

Table S1 msphere.00597-23-s0002.docx List and sequences of primers and gRNAs used in this study.

ASM does not own the copyrights to Supplemental Material that may be linked to, or accessed through, an article. The authors have granted ASM a non-exclusive, world-wide license to publish the Supplemental Material files. Please contact the corresponding author directly for reuse.
