# Supplementary material for: A Toxoplasma gondii putative amino acid transporter localizes to the plant-like vacuolar compartment and controls parasite extracellular survival and stage differentiation
Source: mSphere. 2023 Dec 5;9(1):e00597-23. doi: 10.1128/msphere.00597-23 (PMC10871165; doi:10.1128/msphere.00597-23)
Supplement: Supplemental Figures — Fig. S1 to S10. [file msphere.00597-23-s0001.pdf]

```
Query=
Length=561
                                                                      Score     E
Sequences producing significant alignments:                          (Bits)  Value

TGME49_227430-t26_1-p1 | transcript=TGME49_227430-t26_1 | gene=TG...  41.6    6e-04
TGME49_227580-t26_1-p1 | transcript=TGME49_227580-t26_1 | gene=TG...  39.7    0.002
TGME49_227570-t26_1-p1 | transcript=TGME49_227570-t26_1 | gene=TG...  37.7    0.008


>TGME49_227430-t26_1-p1 | transcript=TGME49_227430-t26_1 | gene=TGME49_227430
| organism=Toxoplasma_gondii_ME49 | gene_product=transmembrane
amino acid transporter protein | transcript_product=transmembrane
amino acid transporter protein | location=TGME49_chrX:941693-946193(+)
| protein_length=665 | sequence_SO=chromosome
| SO=protein_coding_gene | is_pseudo=false
Length=665

 Score = 41.6 bits (96),  Expect = 6e-04, Method: Compositional matrix adjust.
 Identities = 40/146 (27%), Positives = 62/146 (42%), Gaps = 21/146 (14%)

Query  425  IEQNFLDNFPSSDTLSFIARIFLLFQMMTVYPLL----------GYLARVQLLGHIFGDI  474
            +E   L N P+ + L    +    F + T  PL+           +L+R +
Sbjct  510  VEPVVLSNLPA-NILGLGVQAVFAFAIATSVPLMIFPASAIVEHWFLSRFKFRSSTSTVA  568

Query  475  YPSIFHVLILNLIIVGAGVIMACFYPNIGGIIRYSGAACGLAFVFIYPSLIYIISLHQEE  534
              SI  + ++ +I+  A V +      I   +   G ACG    F++P+LI+ I LH +E
Sbjct  569  IYSIVRICLVIIILAAATVGLQ----QIDNFVALIGGACGAPLTFVFPTLIH-IKLHPDE  623

Query  535  RLTWPKLIFHVFIIILGVANLIVQFF  560
               W   +FH FII  GV    VQ F
Sbjct  624  AKRWK--LFHYFIICSGVG---VQVF  644

>TGME49_227580-t26_1-p1 | transcript=TGME49_227580-t26_1 | gene=TGME49_227580
| organism=Toxoplasma_gondii_ME49 | gene_product=transmembrane
amino acid transporter protein | transcript_product=transmembrane
amino acid transporter protein | location=TGME49_chrX:901646-905215(-)
| protein_length=500 | sequence_SO=chromosome
| SO=protein_coding_gene | is_pseudo=false
Length=500

 Score = 39.7 bits (91),  Expect = 0.002, Method: Compositional matrix adjust.
 Identities = 38/162 (23%), Positives = 84/162 (52%), Gaps = 11/162 (7%)

Query  398  MLVTLTYLYIGVLVFASFPSPPLSKDCIEQNFLDNFPSSDTLSFIARIFLLFQMMTVYPL  457
            ++ +LT L +  +V+++       KD +++  L N PS    + I  IF++  +++ YPL
Sbjct  314  LISSLTGLILLYVVYSTICVLAFGKD-VKEIILSNLPSGPLGTTIQAIFVIV-VLSTYPL  371

Query  458  LGY----LARVQLLGHIFGDIYPSI-FHVLILNLIIVGAGVIMACFYPN-IGGIIRYSGA  511
            + Y    +   +LL ++      ++    + + +I+V + + MA    + +GG++   GA
Sbjct  372  MLYPATGIVEERLLPYVVTSCRWALQLASVTIRIILVLSTLAMATLGKHQLGGLVSLIGA  431

Query  512  ACGLAFVFIYPSLIYIISLHQEERLTWPKLIFHVFIIILGVA  553
             CG+   FI+P+L+++     ++ L   + + H  I++ G A
Sbjct  432  VCGVPLAFIFPALMHLKLRRPQQFL---RRLSHYLIVLSGFA  470


>TGME49_227570-t26_1-p1 | transcript=TGME49_227570-t26_1 | gene=TGME49_227570
| organism=Toxoplasma_gondii_ME49 | gene_product=transmembrane
amino acid transporter protein | transcript_product=transmembrane
amino acid transporter protein | location=TGME49_chrX:909219-912881(-)
| protein_length=531 | sequence_SO=chromosome
| SO=protein_coding_gene | is_pseudo=false
Length=531

 Score = 37.7 bits (86),  Expect = 0.008, Method: Compositional matrix adjust.
 Identities = 98/509 (19%), Positives = 192/509 (38%), Gaps = 84/509 (17%)

Query  55   QRVSDHASAMNKRIHYYSRLTTPADKA-LIAPDHVVPAPEECYVYSPLGSAYKLQSYTEG  113
            Q  +D  SA  K   Y +R +T    A  +  D    +     V + +G   + +
Sbjct  70   QAENDEGSARGKYTKYTTRSSTSGGSADCLFDDGDASSINGSVVETNIGVLDEKK-----  124

Query  114  YGKNTSLVTIFMIWNTMMGTSILSIPWGIKQAGFTTGMCVIILMGLLTLYCCYRVVKSRT  173
              K  +  T  +I  + +G  IL +P  + + G    +C+++ +  L LYC + ++K
Sbjct  125  --KTATASTAVVILKSFVGAGILFLPHAVMKGGLIFSLCLLVGVVGLALYCMHVLIKCCE  182

Query  174  MMFSLDTTSWEYPDVCRHYFGSFGQWSSLLFSLVSLIGAMIVYWVLMSNFLFNTGKFIFN  233
                    +  Y ++    FG++G             G  I   V +S   F T   I
Sbjct  183  -----PGAAESYEELGEQAFGAWG-------------GMAIESCVFISQLGFCT---INA  221

Query  234  FIHHINDTDTILSTNNSNPVICPSAGSGGHPDNSSMIFYANDTGAQQFEKWWDKSRTVPF  293
             +   N  D I S    +P          H    ++I             W      +PF
Sbjct  222  AVVAGNLRDVIWSATQCSPDF--------HLPVKALI-------------WCGAILYIPF  260

Query  294  YLVGLLLPLLNFKSPSFFSKFNILGTVSVLYLIFLVTFKAVRLGFHLEFHWFIPTEFFVP  353
             L+         K   + +   ++G +S +  + L+    V +G  +  +  I     V
Sbjct  261  SLI---------KHIKYLAPLMLVGNISTVVGVALLM---VCVGMEVGSNHGINEVDLVN  308

Query  354  EIRFQFPQLTGVLTLAFFIHNCIITLLKNNKKQ-ENNVRDLCIAYML-VTLTYLYIGVLV  411
                 +P + G     +     ++ + K++KK  ++N   L IA ++ + +TY+   V
Sbjct  309  --TSNWPLVLGTSIYLWEGAGMVLPIRKSSKKAVQDNFSKLLIACLVALVITYMVYSVTC  366

Query  412  FASFPSPPLSKDCIEQNFLDNFPSSDTLSFIARIFLLFQMMTVYPLLGYLARVQLLGHIF  471
              +F         +E   L N PS   L    +    F ++  YPL+ + A   +   +
Sbjct  367  NLAFGRH------LEVVILSNLPSG-VLGLSVQTIFAFAVLVTYPLMLFPASTIVEQRLL  419

Query  472  GDIYPS-------IFHVLILNLIIVGAGVIMACFYPNIGGIIRYSGAACGLAFVFIYPSL  524
              +  S       +   + ++L+I+  G+  +     +  ++   G  CG+   FI+P L
Sbjct  420  SVVNVSDRVLNWVVGPTIRISLVILTLGLATSGLQ-QLDNVVALIGGVCGVPLSFIFPVL  478

Query  525  IYIISLHQEERLTWPKLIFHVFIIILGVA  553
            ++ + L  ++R+     + H FI++ G A
Sbjct  479  LH-MKLRGDKRIA--SKLLHWFIVLGGFA  504



Lambda      K        H
   0.329    0.143    0.453

Gapped
Lambda      K        H
   0.267    0.0410    0.140

Effective search space used: 2681193720


  Database: /eupath/data/apiSiteFilesStaging/ToxoDB/49/real/webServices/ToxoDB/r
elease-CURRENT/TgondiiME49/blast/TgondiiME49AnnotatedProteins
    Posted date:  Jun 5, 2023  10:36 AM
  Number of letters in database: 6,669,204
  Number of sequences in database:  8,322
```

Figure S1. Search for putative PLVAC amino acid transporters in *T. gondii*. The Lysosomal transporter SLC38A9 was used as query to interrogate ToxoDB database (https://toxodb.org/toxo/app) to identify amino acid transporters potentially localized to the parasite PLVAC.

A

```
>
TGME49_226060-t26_1-p1
 | transcript=TGME49_226060-t26_1 | gene=TGME49_226060
| organism=Toxoplasma_gondii_ME49 | gene_product=transmembrane
amino acid transporter protein | transcript_product=transmembrane
amino acid transporter protein | location=TGME49_chrX:1836342-1842447(+)
| protein_length=717 |
sequence_SO=chromosome | SO=protein_coding_gene | is_pseudo=false
Length=717

 Score = 27.3 bits (59),  Expect = 15, Method: Compositional matrix adjust.
 Identities = 19/62 (31%), Positives = 28/62 (45%), Gaps = 6/62 (10%)

Query  396  AYMLVTLTYLYIGVLVFASFPSPPLSKDCIEQNFLDNFPSSDTLSFIARIFLLFQMMTVY  455
            A +L    Y  I  L + SF      +   +QNF+ N+ S D L  +  + L F M+
Sbjct  341  AALLEYCLYTPIATLGYLSF------RGVTKQNFMLNYSSEDQLMHVCTLLLSFSMVLGV  394

Query  456  PL  457
            PL
Sbjct  395  PL  396
```

B

```
>
TGME49_226060-t26_1-p1
 | transcript=TGME49_226060-t26_1 | gene=TGME49_226060
| organism=Toxoplasma_gondii_ME49 | gene_product=transmembrane
amino acid transporter protein | transcript_product=transmembrane
amino acid transporter protein | location=TGME49_chrX:1836342-1842447(+)
| protein_length=717 |
sequence_SO=chromosome | SO=protein_coding_gene | is_pseudo=false
Length=717

 Score = 81.6 bits (200),  Expect = 2e-16, Method: Compositional matrix adjust.
 Identities = 76/298 (26%), Positives = 130/298 (44%), Gaps = 25/298 (8%)

Query  3    ATSSALSSTANLVKTIVGAGTLAIPYSFKSDGVLVGVILTLLAAVTSGLGLFVLSKCSKT  62
            A  S   S   L  + +GAG LA PY+ +  G+L+G+ L  +    S    ++L   SK
Sbjct  116  ARGSLRGSVLTLASSCLGAGVLATPYAMQETGLLIGLSLLCMHTFVSFFTTYILMASSKF  175

Query  63   LINPRNSSFFTLCMLTYPILAP-IFDLAMIVQCFGVGLSYLVLIGDLFPG------LF--  113
                 +S++  L     P L     D  +++   GV LS+LV +GD  P       LF
Sbjct  176  F---GSSTYAELAHRASPRLPRRAVDAIIVLNGLGVCLSFLVFLGDFLPASLENLQLFPR  232

Query  114  -GGERNYWIIASAVIIIPLCLVKKLDQLKYSSILGLFALAY-ISILVFSHFVFELGKGEL  171
                R   + AS V+I PL +  +L  L++ +   +  AL + +S +V+
Sbjct  233  ATDHRAALLCASMVVIFPLSVQPRLSALRHFAFFPVCALLFSLSCVVYRSL---------  283

Query  172  TNILRNDICWWKIHDFK-GLLSTFSIIIFAFTGSMNLFPMINELKDNSMENITFVINNSI  230
             ++LR      ++ +       +F++ +FAF   +N+ P+  EL++ +   +  V   +
Sbjct  284  -HLLREQTAPIRLVNLNWNFFKSFNVFLFAFMQHINVCPIGRELQNPTDPRVYKVSLRAA  342

Query  231  SLSTALFLIVGLSGYLTFGNETLGNLMLNYDPNSIWIVIGKFCLGSMLILSFPLLFHP  288
             L   L+  +   GYL+F   T  N MLNY      + +    L   ++L  PL   P
Sbjct  343  LLEYCLYTPIATLGYLSFRGVTKQNFMLNYSSEDQLMHVCTLLLSFSMVLGVPLTLIP  400
```

Figure S2. Identification of the fourth candidate as a potential PLVAC amino acid transporter. A) By adjusting the expectation value in BLAST parameters from the default of 10 to 20, the search for putative PLVAC transporters using SLC38A9 as a query also revealed the presence of the *T. gondii* protein TGME49_226060. B) *Saccharomyces cerevisiae* vacuolar amino acid transporter 7 (Avt7) recognized TGME49_226060 as the best match in ToxoDB.

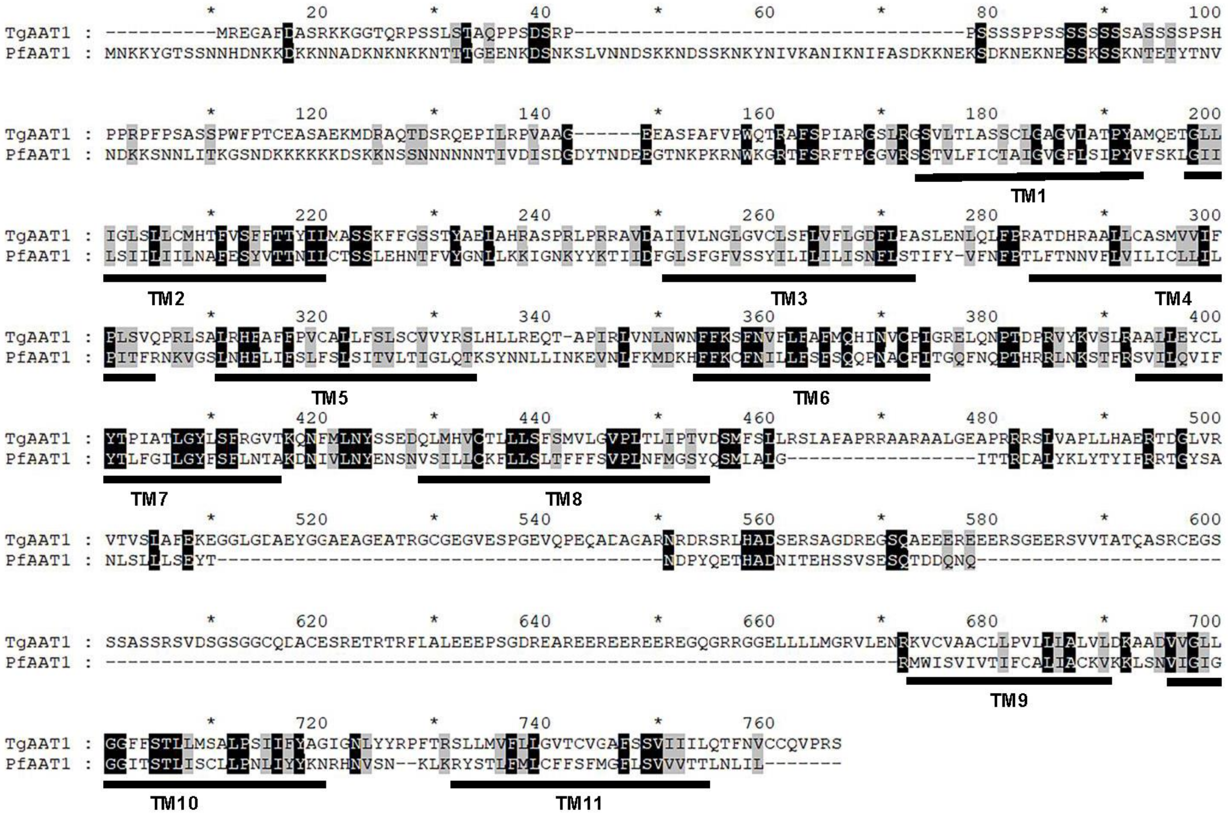

Figure S3. Sequence alignment between TgAAT1 and PfAAT1 using ClustalW software (https://www.genome.jp/tools-bin/clustalw). Transmembrane domains are underlined and number as TM1-11. PlasmoDB (https://plasmodb.org/plasmo/app) accession number of PfAAT1 is PF3D7_0629500.

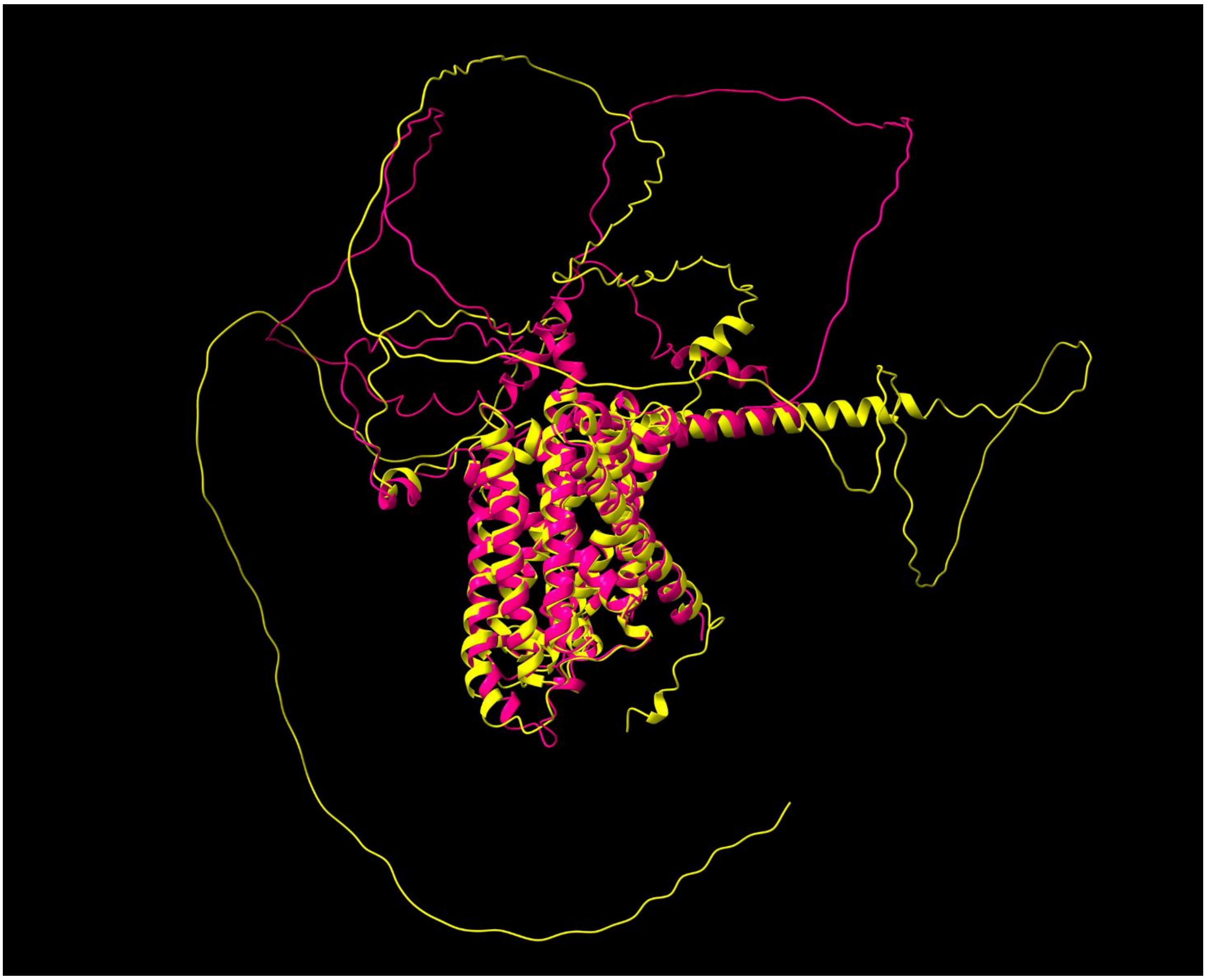

Figure S4. AlphaFold2 (https://alphafold.ebi.ac.uk/) model and structural comparison (http://ekhidna2.biocenter.helsinki.fi/dali/) of PfAAT1 (Pink) and TgAAT1 (Yellow).

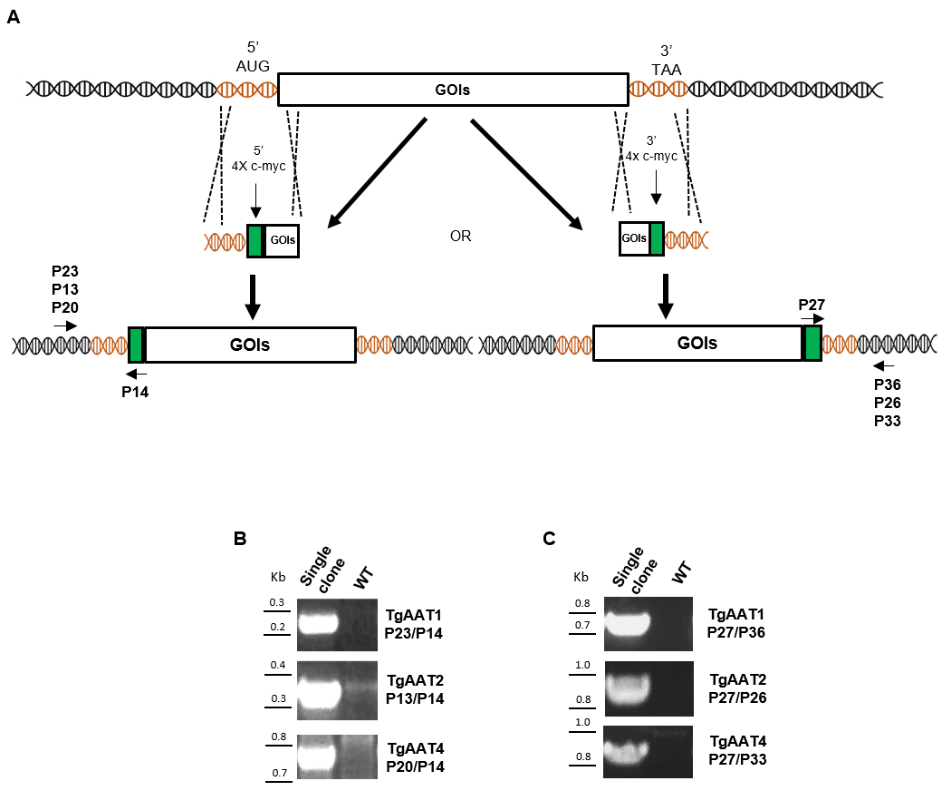

Figure S5. Strategy to endogenously tag TgAATs with 4x c-myc epitopes. A) A plasmid carrying the sequence encoding 4xc-myc epitopes was used to generate ~2μg of PCR fragment composed of the tag sequence flanked at both sides by 40 bp of homology to regions immediately adjacent to the sequences coding either the N-terminal (left panel) or C-terminal (right panel) regions of the TgAAT proteins. Integration of the 4x c-myc tag in the selected place by double-crossover homologous recombination was induced by Cas9 cleavage directed by a specific gRNA. Validation of correct epitope sequence integration was validated by PCR analysis. Panel B shows an agarose electrophoresis gel of the PCR screening products of single clones tagged at the N-terminus. The same analysis was employed for the C-terminus tagging (panel C). All primer sequences and gRNAs are reported in Table S1.

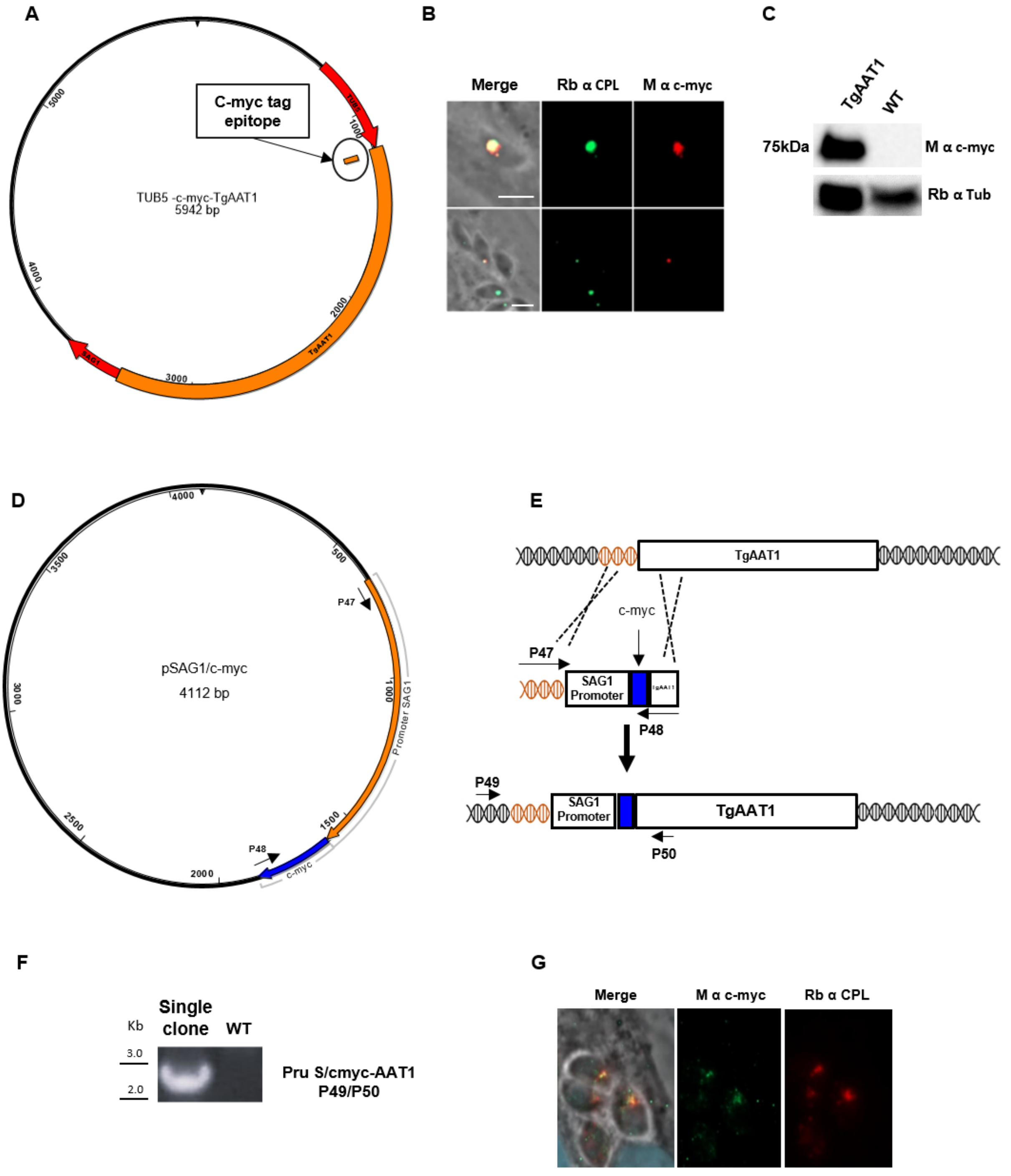

Figure S6. Panels A-C. Transient overexpression of TgAAT1. A) Map of the plasmid generated to overexpress TgAAT1 fused at its N-terminus to a single copy c-myc epitope. B) Representative images of parasites transiently transfected with the TgAAT1 overexpressing vector and processed for IFA using anti-c-myc (red) and anti-TgCPL (green) antibodies. Scale bar 10µm. C) WB analysis of the protein extract of parasites transiently transfected with the TgAAT1 overexpressing vector. The filter was first probed with mouse anti-c-myc and then with rabit anti-tubulin as a loading

control. Panels D-G. Replacement of the TgAAT1 promoter with that of the TgSAG1 gene enabled the visualization of TgAAT1 through immunofluorescence assay (IFA). D) Map of the plasmid used to amplify the repair template consisting of the TgSAG1-c-myc cassette (TgSAG1 promoter transcribing the sequence encoding the c-myc epitope fused to the N-terminus of a gene of interest, GOI) flanked on both sides by 40 bp of homology to regions immediately adjacent to the TgAAT1 promoter. Primers and gRNAs used are reported in Table S1. E) Schematic representation of the strategy used to replace the TgAAT1 promoter with that of TgSAG1 gene. F) PCR validation of correct replacement of the TgAAT1 promoter with that of the TgSAG1 gene in a single clone. G) Representative images of parasites expressing TgAAT1 under the TgSAG1 promoter processed for IFA using anti-c-myc (red) and anti-TgCPL (green) antibodies. Scale bar 5μm.

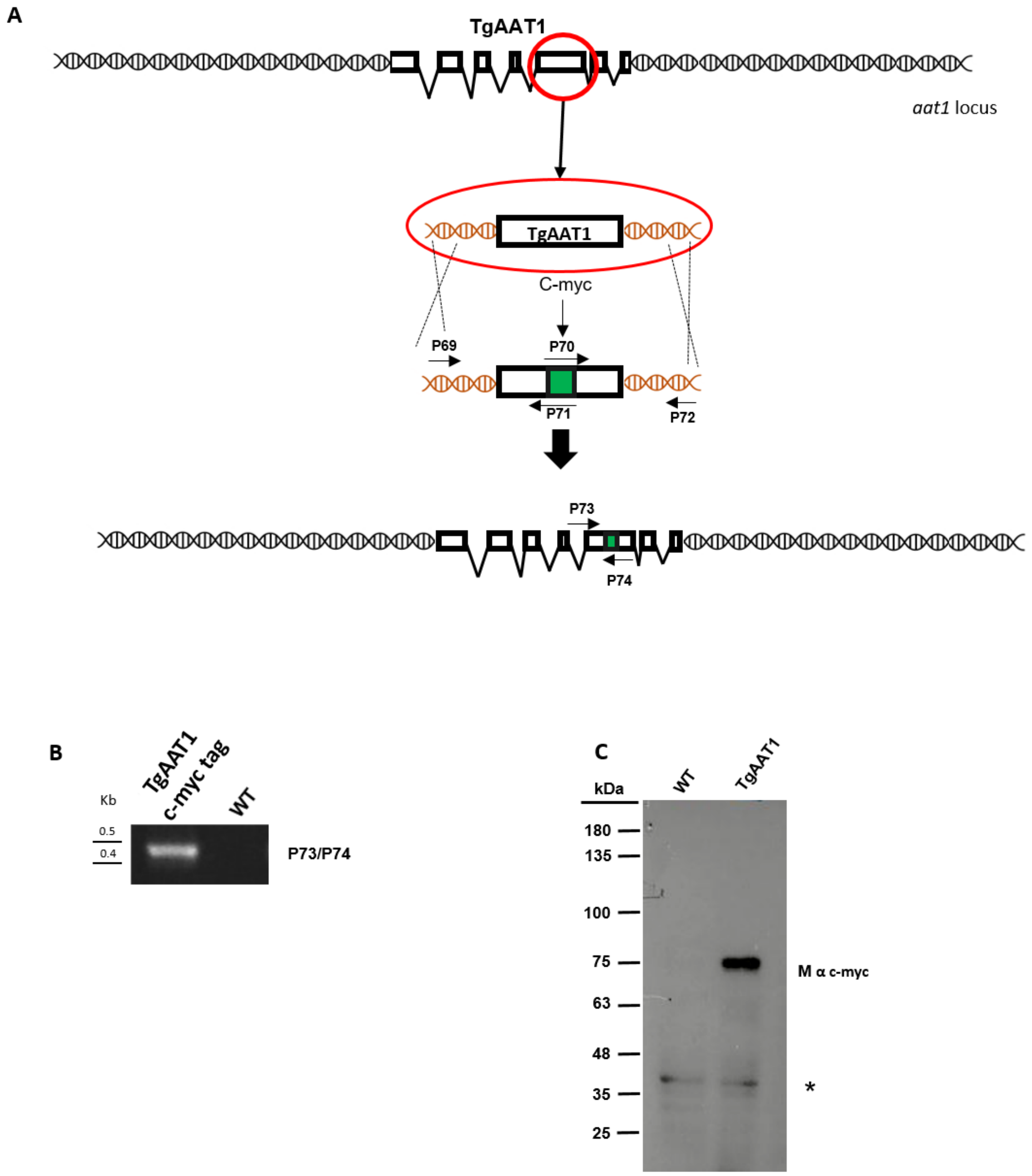

Figure S7. C-myc internal tagging of TgAAT1. A) Schematic representation of the strategy to internally tag TgAAT1. Overlap PCR were employed to generate a repair template consisting of the sequence encoding the c-myc epitope flanked at both sides by homology to regions immediately adjacent to the nucleotide triplet encoding the residue 508 of the TgAAT1 protein. Primers P69/P70 and P71/P72 were used to generate the two PCR products that were fused by overlap PCR using primers P69/P72 to create the repair template. B) PCR validation to confirm the successful

insertion of the c-myc coding sequence in the intended place within the TgAAT1 gene in the Pru strain. All primer sequences and gRNAs are reported in Table S1. D) Western blot analysis of the protein extract of internally tagged TgAAT1 parasites. Proteins were extracted from $5x10^6$ TgAAT1-tagged and parental parasites using RIPA buffer. They were then separated on a 10% acrylamide gel and subsequently transferred to a filter. The filter was probed with mouse anti-c-myc antibodies. The star indicates the presence of a non-specific band on both the WT sample and the c-myc internal tagging of TgAAT1.

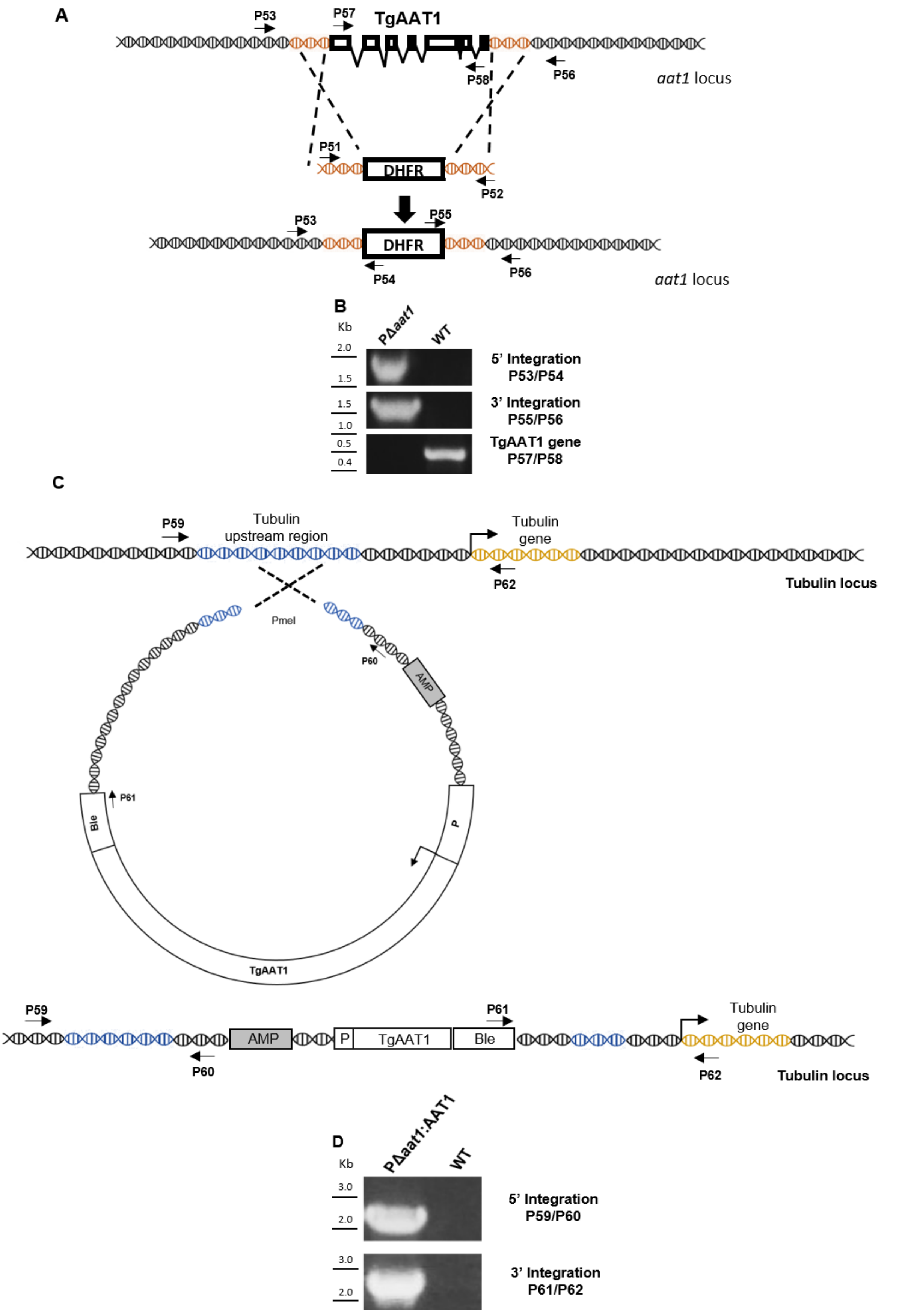

Figure S8. Panels A-B. Generation of the PΔ*aat1* strain. A) Schematic representation of the strategy used to delete the TgAAT1 gene. To generate TgAAT1 knockout parasites, two different

gRNAs were used for Cas9 to cleave at both the start and stop codon *TgAAT1*. A repair template composed of the DHFR selection cassette flanked at both sides by 40 bp of homology to regions immediately adjacent to the start and stop codon of the TgAAT1 gene was used to select knockout parasites resistant to pyrimethamine selection. B) PCR validation to confirm the successful deletion of the TgAAT1 gene in the generated knockout parasites.
Panels C-D. Creation and validation of genetically PΔ*aat1*:AAT1 complemented parasites. C) Schematics of single crossover integration to restore expression of TgAAT1 via genetic complementation with constructs containing a bleomycin selection cassette (BLEO). Single crossover integration was directed to the tubulin locus via homologous regions indicated in blue. Primers used to assess integration of the complementation construct are shown as numbered arrows. D) PCR analysis of integration at the tubulin locus using primers shown in C). All primer sequences and gRNAs are reported in Table S1.

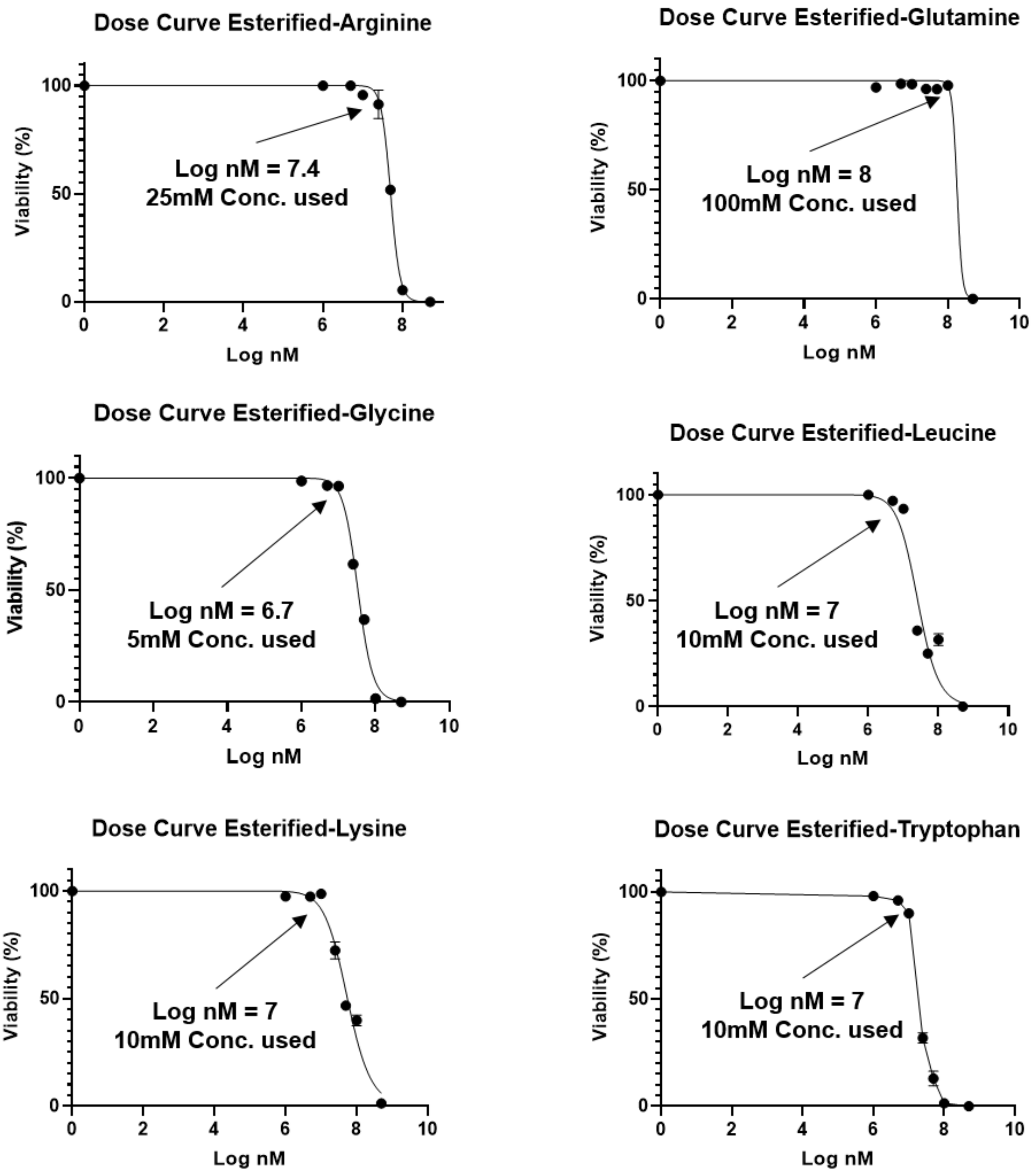

Figure S9. Dose-Response Curves of Esterified Amino Acids. Each esterified amino acid was subjected to dose-response analysis to determine the concentration range that does not affect the viability of the Pru strain. Extracellular Pru tachyzoites were incubated in either normal RPMI media or RPMI media supplemented with one of six different esterified amino acids at varying concentrations (0, 1mM, 5mM, 10mM, 25mM, 50mM, 100mM, 500mM) for 1 h at 37°C. Viability was assessed using the PMA/qPCR method. Error bars represent the standard deviation from two independent experiments.

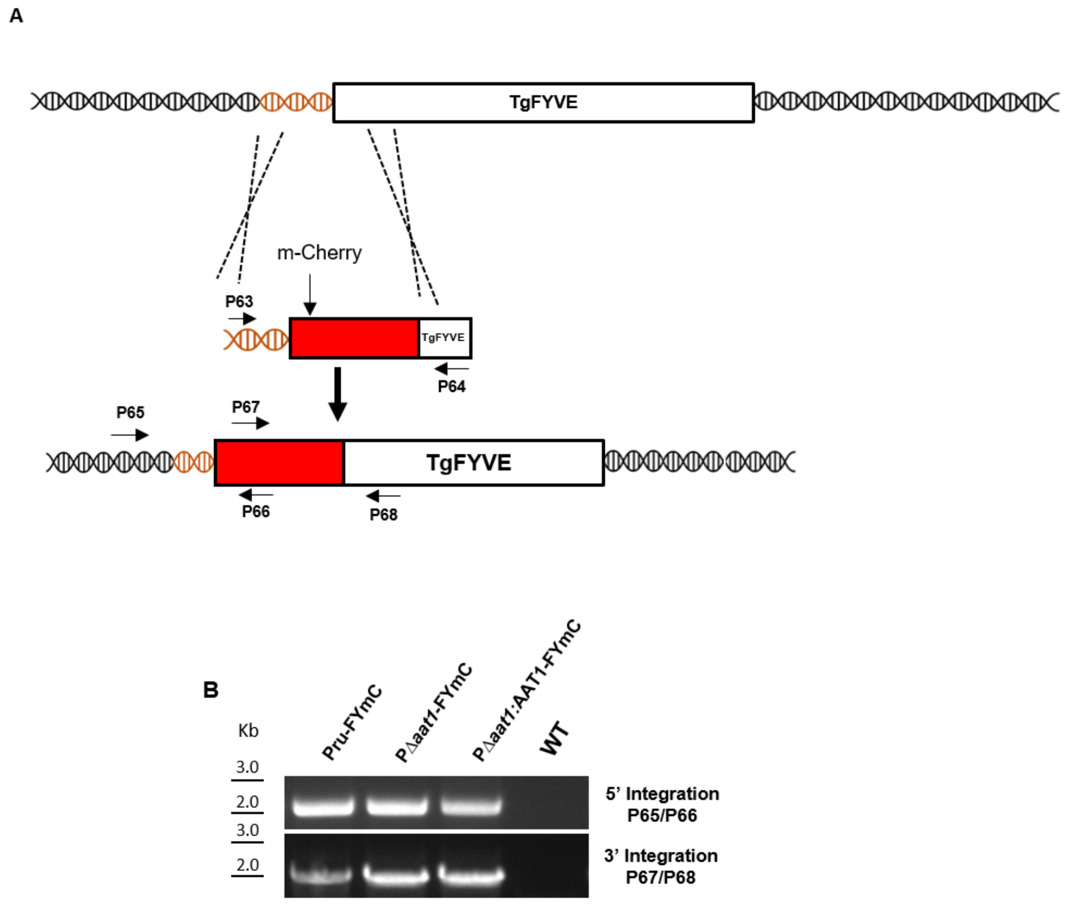

Figure S10. N-terminus tagging of TgFYVE with mCherry in Pru, PΔ*aat1* and PΔ*aat1:AAT1*. A) Schematic representation of the strategy to tag TgFYVE with mCherry. The repair template used to generate this strain contained the mCherry coding sequence flanked by 40 bp homology to the sequence upstream and downstream the start codon of the TgFYVE gene. Integration of the repair template in the intended place was achieved co-transfecting parasites with a plasmid expressing Cas9 and a gRNA that guided the nuclease to cleave close the start codon of the TgFYVE gene. B) PCR validation to confirm the successful integration of the mCherry coding sequence at the start codon of the TgFYVE gene in Pru, PΔ*aat1* and PΔ*aat1:AAT1* parasites. All primer sequences and gRNAs are reported in Table S1.
