# Supplementary material for: A Toxoplasma gondii putative amino acid transporter localizes to the plant-like vacuolar compartment and controls parasite extracellular survival and stage differentiation
Source: mSphere. 2023 Dec 5;9(1):e00597-23. doi: 10.1128/msphere.00597-23 (PMC10871165; doi:10.1128/msphere.00597-23)
Supplement: Table S1 — List and sequences of primers and gRNAs used in this study. [file msphere.00597-23-s0002.docx]

**Supplementary Table S1:** List and sequences of primers and gRNAs used in this study.

| Primer Name | Primer Description | Forward Primer  (5’- 3’) | | Reverse Primer  (5’-3’) | Product  Size (bp) | |  |
| --- | --- | --- | --- | --- | --- | --- | --- |
| rtPCR (Fig. S7) | | | | | | | |
| P1/P2 | TgAAT2 cDNA amplification | CAGATAGGACAGATGCCGAGGATGAC | | CTTTCCCTCTACTTGCCGAACTGACAG | 362 | |  |
| P3/P4 | TgAAT3 cDNA amplification | CAAGCAGTATCAACGGGTCGGTTGTAG | | CTACTGTGCTGATGTTGCCTACAAGC | 532 | |  |
| P5/P6 | TgAAT4 cDNA amplification | CCGAGATTATTTTCTCGCAACGAAGAC | | CGGTGACTAGAATGACATCGCGTAG | 401 | |  |
| P7/P8 | α-Tub | GCGTCTTCTTGGATTTGGAG | | TGGAGACCAGTGCAGTTGTC | 206 | |  |
| P9/P10 | TgAAT1 cDNA amplification | ATGCGTGAGGGTGCGTTCGACGCCTC | | CTAGTGCAGCACAGACCGCGGGACCTG | 2154 | |  |
| Endogenous Tags 4x c-myc N-T TgAATs (Fig. S8) | | | | | | | |
| gRNA1 | gRNA TgAAT2 N-T tag | UAACGUCGCUUCUUGACAUC | |  |  | |  |
| P11/P12 | Amplification 4xc-myc to tag N-T TgAAT2 | GTAGTAGGAGGTAACTGCCCGAAGTAGTTCTTCGTCGATGGCATTGGAGCAGAAGCTAATTAGC | | GCCAATGAAATGCCGAATTGGTGAAGGGCCTCTCTAACGTCGCTTCTTGAGTGATTAATTAAGTACAGATCCTCTTCTG | 230 | |  |
| P13/P14 | Test Integration TgAAT2 N-T 4xc-myc tag | CACATAACAGACAGTGAAACCTTG | | AAGGTCCTCCTCGGAGATGAGCTTCTG | 310 | |  |
| gRNA2 | gRNA TgAAT4 N-T tag | UUCGUGUCGAUUUCCCUGUC | |  |  | |  |
| P18/P19 | Amplification 4xc-myc to tag N-T TgAAT4 | TGCTTCCCGTTGCTTTTGTCTGTCTGCAGAGCCAGAGATGGCATTGGAGCAGAAGCTAATTAGC | | CAGAGACTCCAGGACTGGAGTGACCCGATTCGATTGCCATGTGATTAATTAAGTACAGATCCTCTTCTG | 221 | |  |
| P20/P14 | Test Integration TgAAT4 N-T 4xc-myc tag | GTCATCGCGACGCCGTACGGTCATG | | AAGGTCCTCCTCGGAGATGAGCTTCTG | 783 | |  |
| gRNA3 | gRNA TgAAT1 N-T tag | GGCUCUAAAAGAGCUCGCGC | |  |  | |  |
| P21/P22 | Amplification 4xc-myc to tag N-T TgAAT1 | CCTTTTCTCGCCTCGGCCCAGGCTCTAAAAGAGCTCGCGCAGGATGGAGCAGAAGCTAATTAGC | | CAGAAGAGGATCTGTA CGTGAGGGTGCGTTCGACGCCTCCCGAAAAAAGGGAGGA | 207 | |  |
| P23/P14 | Test Integration TgAAT1 N-T 4xc-myc tag | GCGCTCTTCGTCGACGAAGGCAG | | AAGGTCCTCCTCGGAGATGAGCTTCTG | 263 | |  |
| gRNA4 | gRNA TgAAT2 C-T tag | CUGCGAAUCGGCACUGGCGU | |  |  | |  |
| P24/P25 | Amplification 4xc-myc to tag C-T TgAAT2 | ATCGACAGTGGCAATCTACAGCATTGTGCGGATATGCTTG GAGCAGAAGCTAATTAGC | | CAGAAGAGGATCTGTATAGACCATTCATGCACGATTTATTGGCGAATCATGCGTCC | 202 | |  |
| P27/P26 | Test Integration TgAAT2 C-T 4xc-myc tag | AAGGTCCTCCTCGGAGATGAGCTTCTG | | GCATCGGCGATCGTTGAGCATTGG | 900 | |  |
| gRNA5 | gRNA TgAAT4 C-T tag | AAGGUGCGCCGAUUCAACAC | |  |  | |  |
| P31/P32 | Amplification 4xc-myc to tag C-T TgAAT4 | TGACTACGGCAGCGCTTGCCCAAATGTGAATCTAAGCGACGAGCAGAAGCTAATTAGC | | CAGAAGAGGATCTGTATAACAGGAGAAAAGTAGCGCATGTCACGTGGATTGACTGGGTC | 205 | |  |
| P27/P33 | Test Integration TgAAT4 C-T 4xc-myc tag | AAGGTCCTCCTCGGAGATGAGCTTCTG | | TACCGGGCCCTCTGCCAGGCAATCTTCGTCATTG | 830 | |  |
| gRNA6 | gRNA TgAAT1 C-T tag | CCGUCGGGCCUCCAGCGUGG | |  |  | |  |
| P34/P35 | Amplification 4xc-myc to tag C-T TgAAT1 | CTTCAATGTCTGCTGCCAGGTCCCGCGGTCTGTGCTGCACGAGCAGAAGCTAATTAGC | | CAGAAGAGGATCTGTATAGGTCTGGAGGGGGAACACTCCGAGAAGAATGCTGGCGA | 207 | |  |
| P27/P36 | Test Integration TgAAT1 C-T 4xc-myc tag | AAGGTCCTCCTCGGAGATGAGCTTCTG | | CATGGTCTTCCTTCTCGGCGTGACTTG | 715 | |  |
| Transient overexpression TgAATs (Fig. S9) | | | | | | | |
| P37 | c-myc tag for AAT1 | CGTTGGCATTTTTTCTTGAATTCCCTTTTTCGACAAAATGAGAGAATGCAGGGAGAGCAGAAGCTAATTAGCGAGGAGGACCTGCGTGAGGGTGCGTTCGACGCC | |  |  | |  |
| P39 | c-myc tag for AAT2 | CGTTGGCATTTTTTCTTGAATTCCCTTTTTCGACAAAATGAGAGAATGCAGGGAGAGCAGAAGCTAATTAGCGAGGAGGACCTGTCAAGAAGCGACGTTAGGGAGGCC | |  |  | |  |
| P41 | c-myc tag for AAT3 | CGTTGGCATTTTTTCTTGAATTCCCTTTTTCGACAAAATGAGAGAATGCAGGGAGAGCAGAAGCTAATTAGCGAGGAGGACCTGGAAGTGACGGCTCAGCGGGTTTGT | |  |  | |  |
| P43 | c-myc tag for AAT4 | CGTTGGCATTTTTTCTTGAATTCCCTTTTTCGACAAAATGAGAGAATGCAGGGAGAGCAGAAGCTAATTAGCGAGGAGGACCTGATGATGGCAATCGAATCGGGTCAC | |  |  | |  |
| SAG1-c-myc-AAT1 (Fig. S10) | | | | | | | |
| gRNA3 | gRNA TgAAT1 N-T | GGCUCUAAAAGAGCUCGCGC | |  |  | |  |
| P45/P46 | Sag1 promoter cassette | ATTGGGTACCTATGTCACCATGAAGCAGATCGTCAG | | CAGAAGCTAATTAGCGAGGAGGACCTGCGTGAGGGTGCGTTCGACGCCTCCCGAAAAAAGGGAGGAAC | 920 | |  |
| P47/P48 | Repair template SAG1-c-myc-AAT1 | CGCCGTCTCTCCAGCCGGTCTGAGAGTGTGCGTCTCCGGCCTATGTCACCATGAAGCAGATCGTCAG | | CAAGCAGGAGGAAGCCAGCGT | 1400 | |  |
| P49/P50 | 5’ Integration SAG1-c-myc-AAT1 | GATACAGGCAAGGCGCCGATCAGGTG | | AAGGGAATTCTAATGAAGTGGTGCAGCGAAACCGAC | 2613 | |  |
| TgAAT1 Knockout generation (Fig. S11) | | | | | | | |
| gRNA3 | gRNA TgAAT1 N-T | GGCUCUAAAAGAGCUCGCGC | |  |  | |  |
| gRNA6 | gRNA TgAAT1 C-T | CCGUCGGGCCUCCAGCGUGG | |  |  | |  |
| P51/P52 | Repair Template DHFR for TgAAT1 KO | GTTTCCTCTAAAGAAGAAAAGAGACGCAAGGAGGCGAGGTCGACGGTATCGATAAGCTTG | | AGAAATCCAGAAAAACGGTAAATCGACGCCTTTCCGGTGGCGGCCGCTCTAGAACTAGTG | 2710 | |  |
| P53/P54 | 5’ Integration DHFR | GATGGCTGTCTAGTTAATTAATCACCG | | CGCCACAGAGATGCCGACCTTCGCAC | 1600 | |  |
| P55/P56 | 3’ Integration DHFR | CCAGCCGGTCTGAGAGTGTGCGTCTC | | CGCGACGACCAGACACACCGGTTTATGC | 1500 | |  |
| P57/P58 | Check AAT1 Gene deletion | CCAGCCGGTCTGAGAGTGTGCGTCTC | | CAGTCTCCTGCATCGCGTACGGCGTC | 500 | |  |
| TgAAT1 Complementation (Fig. S12) | | | | | | | |
| P59/P60 | 5’ Complement Integration | CAGCACGTGACTCGATGTTTACCGCTGTC | | CACAGGAAACAGCTATGACCATGATTAC | 2000 | |  |
| P61/P62 | 3’ Complement Integration | GACTACGACGAAAGTGATGCGCAGGC | | CCTGGCCGACGTGGATGCTGATAACCTC | 2100 | |  |
| TgFYVE m-Cherry Tag (Fig. S13) | | | | | | | |
| gRNA7 | gRNA TgFYVE N-T | GGGCGAGACAGGCAUCUUUG | |  |  | |  |
| P63/P64 | Repair Template m-Cherry tag | CTCAGACCTTTCAGGCCCTGTACACACCCGCCGCAAAGATGGTGAGCAAGGGCGAGGAGGATAACATG | | GGGAGACCTCTGGAGGGTCGGAAGCCGAGGGCGAGACAGGTCCGGATCCTCCACCCTTGTACAGCTCGTCCATGCCGCC | 800 | |  |
| P65/P66 | 5’ Tag integration | GCAACTCCAAACGCCTCGGCTCTGAG | | GCATGAACTCCTTGATGATGGCCATG | 315 | |  |
| P67/P68 | 3’ Tag integration | GAGGACTACACCATCGTGGAACAGTAC | | GTCGACGTTTCGGCCTGCGTCAGGTTC | 487 | |  |
| TgAAT1 c-myc Tag (Fig. S14) | | | | | | | |
| gRNA8 | gRNA TgAAT1 | GCCGACAGCGAGCGCAGUGC | |  |  | |  |
| P69/P70 | Overlap PCR | GAAGACCAGCTGATGCATGTCTGCAC | | CGCTCCAGGTCCTCCTCGCTAATTAGCTTCTGCTCGCTGTCGGCATGCAGGCGGCTTCGGTC | 440 | |  |
| P71/72 | Overlap PCR | ACAGCGAGCAGAAGCTAATTAGCGAGGAGGACCTGGAGCGCAGTGCGGGGGACCGAGAAGG | | CTTCCTCCAGTGCGAGGAAACGCGTTCTC | 252 | |  |
| P73/P74 | 5’ Tag integration | GAAGACCAGCTGATGCATGTCTGCAC | | GTCCTCCTCGCTAATTAGCTTCTGCTC | 432 | |  |
| Primers used in qPCR | | | | | | |  |
| Tub-F1/ DHFR-R1 | Tubulin promoter/ DHFR cDNA | | GTGTTACACAATCACCTTGTGTGAAG | CGCGACGACCAGACACACCGGTTTATGC | | 119 |  |
| Tx9/Tx11 | Viability assay  Multi-locus target | | AGGAGAGATATCAGGACTGTAG | GCGTCGTCTCGTCTAGATCG | | 165 |  |
